# Coordinative Ring‐Opening Copolymerization of Limonene Carbamate and *ε*‐Caprolactone Toward Phosgene‐ and Isocyanate‐Free Polyesterurethane Block‐Copolymers with Tunable Properties

**DOI:** 10.1002/marc.202500817

**Published:** 2025-11-29

**Authors:** Jonas Futter, Hendrik Pfaadt, Bernhard Rieger

**Affiliations:** ^1^ Wacker‐Lehrstuhl für Makromolekulare Chemie Catalysis Research Center TUM School of Natural Sciences Technische Universität München Garching bei München Germany

**Keywords:** block‐copolymerization, isocyanate‐free synthesis, phosgene‐free synthesis, polyesterurethane, ring‐opening copolymerization

## Abstract

Polyesterurethanes are versatile polymers widely utilized in applications such as foams and adhesives, yet their industrial production relies on toxic and carcinogenic diisocyanates. To address this, isocyanate‐ and phosgene‐free synthetic methods have been explored, with ring‐opening polymerization of cyclic carbamates emerging as a promising alternative. This study presents the coordinative ring‐opening copolymerization of limonene‐based cyclic carbamates with *ε*‐caprolactone to synthesize AB‐block polyesterurethanes. Using the presented method, tunable block copolymer compositions were achieved, verified by NMR, GPC, and FT‐IR analyses. Thermal and optical characterizations by DSC and UV–vis revealed an adjustable glass transition temperature between −9°C and −59°C and transmittance up to 84% for **PLU‐b‐PCL (49:51)**, while tensile testing demonstrated customizable mechanical properties. Notably, **PLU‐b‐PCL (5:95)** exhibited an elongation at break of 582%. These findings provide a basis for sustainable polyesterurethane synthesis by ring‐opening copolymerization and demonstrate the versatility of this method.

## Graphical abstract

This study presents a sustainable, isocyanate‐ and phosgene‐free route to polyesterurethanes via coordinative ring‐opening copolymerization of limonene carbamate and *ε*‐caprolactone. The resulting block copolymers show tunable thermal, optical, and mechanical properties, offering a green alternative for advanced polyurethane materials.

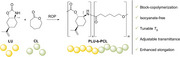

## 1 Introduction

Polyesterurethanes (PU) are highly versatile polymers used in a variety of applications, including foams, coatings, and adhesives, due to their outstanding properties such as flexibility, abrasion resistance, and chemical resistance [1, 2, 3, 4, 5]. The industrial production of PU is based on the polyaddition of polyesterpolyols and diisocyanates. The latter are highly toxic and carcinogenic, which is why they should be avoided in the long term [6, 7, 8]. As a result, several methods for the phosgene‐ and isocyanate‐free synthesis of PU have been developed in recent decades. Various polycondensation processes have been described using polychloroformates, polycarbamates, and other derivatives as isocyanate surrogates. However, these compounds are most commonly produced with phosgene, and stoichiometric amounts of often hazardous by‐products are formed during polymerization [1, 9]. Other polycondensation routes, such as the dehydrogenative coupling of formamides and alcohols or the reaction of diamines, carbon dioxide, and dibromoalkanes, are only suitable for specific, activated substrates [10, 11]. Besides the polyaddition of diamines to polycyclic carbonates, the ring‐opening polymerization (ROP) of cyclic carbamates represents a promising alternative for the sustainable synthesis of PU, as no by‐products are formed during polymerization and the deployed monomers can be derived from carbon dioxide or dimethyl carbonate (DMC) [1, 12, 13, 14, 15, 16]. Although ROP potentially represents a phosgene‐ and isocyanate‐free alternative to industrial polyurethane synthesis, only a few examples are reported in the literature [17, 18, 19, 20]. After Höcker et al. and Thomas et al. reported on the cationic and anionic ROP of cyclic carbamates, we recently presented the coordinative ROP of a limonene‐based carbamate in the presence of Sn(Oct)_2_ [17, 20, 21]. Due to its high glass transition temperature and crystallinity, polylimoneneurethane (**PLU**) qualifies as a suitable hard segment for the synthesis of copolymers. So far, only Höcker and coworkers have reported on the cationic ring‐opening copolymerization (ROCOP) of carbamates and ethers [22]. However, the ROCOP of cyclic carbamates and lactones remains unexplored to date. To expand the potential of this method, we herein present the coordinative ROCOP for the synthesis of block polyesterurethanes.

## 2 Results and Discussion

The synthesis and polymerization of limonene carbamate (**LU**) were carried out according to the procedures our group recently reported [21]. Here, (*R*)‐limonene is selectively epoxidized to *cis*‐limonene (**1**) in the presence of Jacobsen's (*R*,*R*)‐Mn(III) catalyst with *m*‐CPBA, followed by stereoselective *S*_N_1 ring opening with aqueous ammonia to give the amino alcohol (**2**). The cyclic carbamate **LU** is then obtained by reaction with DMC as a sustainable phosgene surrogate (see Scheme S1). Detailed procedures and analytical data for the monomer synthesis are provided in the Supporting Information (see Figures S1–S10). For the copolymerization of **LU** and *ε*‐caprolactone (**CL**), the synthesis of random copolymers was considered in addition to **PLU‐b‐PCL** block copolymers.

However, the polymerization of an equimolar mixture of **LU** and **CL** did not yield a uniform diffusion coefficient in the diffusion‐ordered NMR (DOSY‐NMR) spectrum, and only low conversions were observed (see Figure S25). Attempts to polymerize **CL** first and subsequently add **LU** resulted in low incorporation of **LU** based on ^1^H NMR analysis. This is probably due to the higher nucleophilicity of the amine end group compared to the alcohol end group, which is formed by the ring opening of **LU** and **CL**, respectively [23, 24].

**SCHEME 1:**
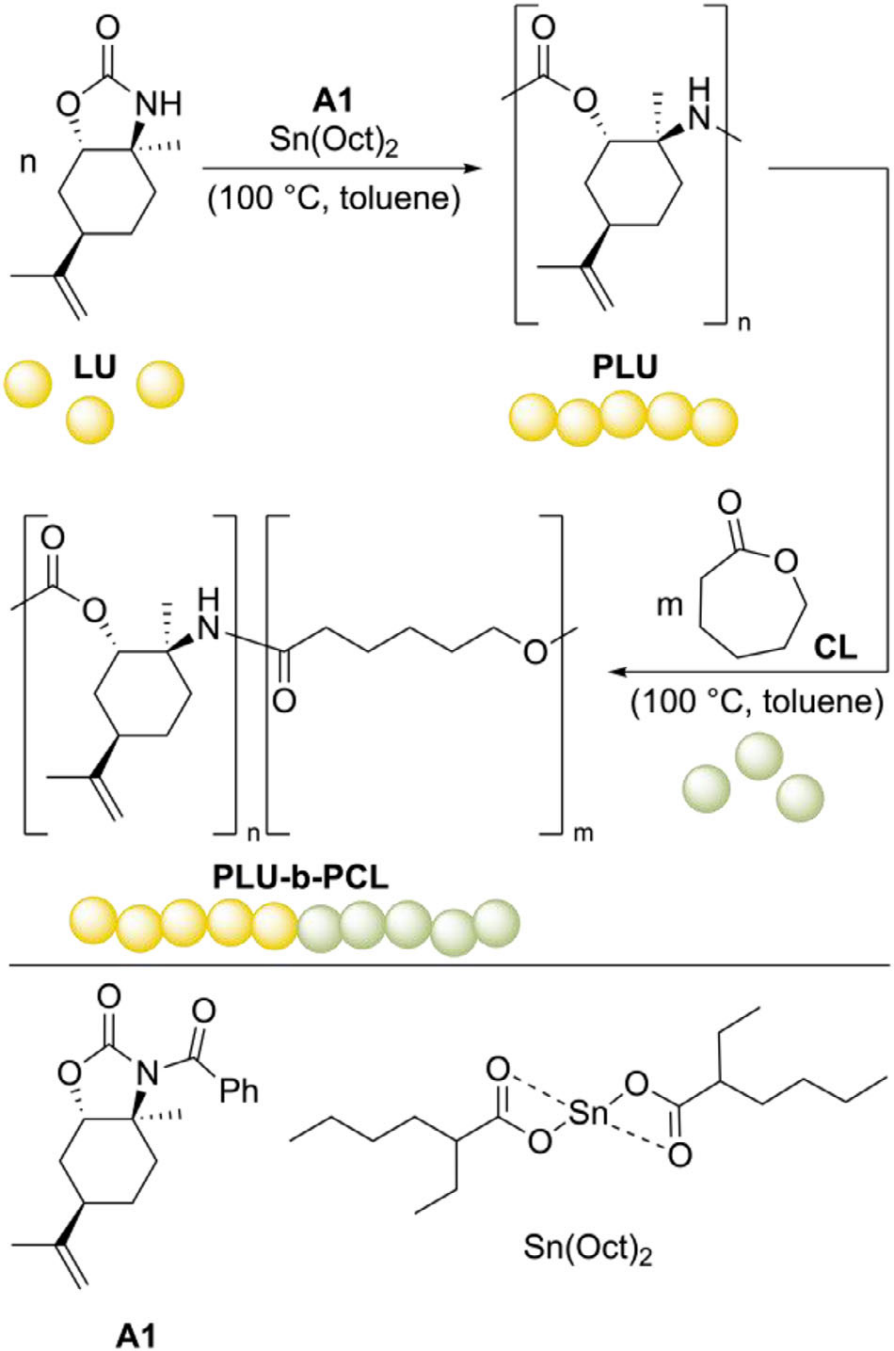
Block ROCOP of **LU** and **CL** using Sn(Oct)_2_ as a catalyst to afford **PLU‐b‐PCL**.

In addition, urethane linkages are generally less susceptible to alcoholysis and aminolysis than ester linkages due to their amide resonance [25]. Based on these results, we focused on first synthesizing the **PLU** block, followed by the **PCL** block (Scheme 1). A ratio of **LU**/**A1**/Sn(Oct)_2_ of 50:1:1 was consistently applied for the **PLU** block, and the reaction time was set to 12 h based on kinetics studies for homopolymerization (see Figure S17). The conversion of **LU** in wet deuterated chloroform was determined by taking an aliquot of the reaction solution. Then a 1 m solution of **CL** was added to achieve the desired composition of **PLU** to **PCL**.

**FIGURE 1:**
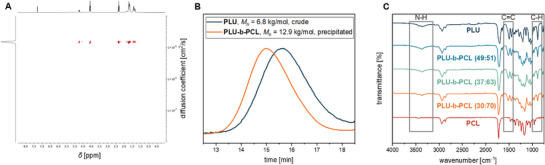
(A) Representative DOSY‐NMR spectra of the **PLU‐b‐PCL (30:70)** (Table 1 entry 6). (B) GPC traces of the crude **PLU** block (blue curve) before **CL** addition and the **PLU‐b‐PCL** copolymer (orange curve) after precipitation from THF with *n*‐pentane (Table 1 entry 4). (C) FT‐IR spectra of **PLU**, various selected **PLU‐b‐PCL**, and **PCL** samples.

Figure 1A shows a representative DOSY NMR spectrum for a **PLU**/**PCL** composition of 30:70 (Table 1 entry 6). The uniform diffusion coefficient of about 1 × 10^−5^ cm^2^/s for the copolymer's proton signals confirms the successful synthesis of polyester urethane **PLU‐b‐PCL**. Further DOSY NMR spectra for the **PLU‐b‐PCL** copolymers with other compositions are listed in the Supporting Information (see Figures S18–S23). All **PLU‐b‐PCL** copolymers produced show a deviating diffusion coefficient compared to the homopolymers **PLU** and **PCL**. In addition, the DOSY‐NMR spectrum of a **PLU**/**PCL** blend shows a non‐uniform diffusion coefficient (see Figure S24). GPC measurements of the individual blocks can also monitor the proceeding of the successful copolymerization. As illustrated in Figure 1B, **PLU‐b‐PCL (30:70)** is eluted after a shorter retention time compared to the **PLU** block of the reaction control, whereby the average molecular weight *M*_n_ increases from 6.8 to 12.9 kg/mol (orange and blue curves, respectively). The monomodal distribution is maintained, and dispersity *Đ* remains in the range of 1.9, suggesting a more uniform ROP of the **PCL** block than that of **PLU**. This assumption agrees with the low dispersities *Đ* in the range of 1.1–1.3 for the ROP of **CL** in the presence of Sn(Oct)_2,_ with the values reported in the literature [26, 27, 28]. In addition, the dispersities of the copolymers are in the range of the **PLU** homopolymer [21]. The composition of the purified **PLU‐b‐PCL** copolymers was determined by integrating the isolated, slightly downfield‐shifted signals of the vinyl and *α*‐protons to the ester moiety in the ^1^H NMR spectrum at *δ* = 4.71 ppm and 4.06 ppm for **PLU** and **PCL**, respectively (see Figure S15). In Table 1, the copolymer compositions are in excellent agreement with the **LU**/**CL** equivalents of the monomers employed. Uniformly for all copolymers, the proportion of **PCL** is somewhat higher than targeted. This can be explained by taking an aliquot of the first synthesized **PLU** block for reaction control via ^1^H NMR and GPC measurements, and the slightly higher **CL** conversion than **LU** (see Figure S17).

**TABLE 1:** Copolymerization of **LU** and **CL** to yield **PLU‐b‐PCL** in various compositions.

	LU/CL^a^	t_Pol_	PLU‐b‐PCL^b^	*M*_n_ ^c^	*M*_w_ ^c^			*M*_n,theo_ ^e^	***T***_g_	***T***_m_
Entry	(%)	(h)	[PLU:PCL]	(kg/mol)	(kg/mol)	*Đ* ^c^	DP^d^	(kg/mol)	(°C)	(°C)
1	67:33	15	65:35	8.4	16.3	2.0	50	10.6	n.d.	n.d.
2	50:50	15	48:52	10.1	19.8	2.0	66	13.3	−9	—
3	44:56	15	42:58	12.0	22.8	1.9	81	15.4	n.d.	n.d.
4	40:60	16	37:63	12.9	25.2	2.0	89	16.3	−16	44
5	36:64	16	33:67	13.2	24.1	1.8	94	17.3	−30	44
6	33:67	16	30:70	14.4	27.6	1.9	104	18.8	−49	47
7^f^	50:50	15	49:51	9.9	20.5	2.1	64	13.5	n.d.	n.d.
8^f^	40:60	16	38:62	12.3	23.4	1.9	85	16.7	n.d.	n.d.
9^f^	33:67	16	31:69	14.5	24.7	2.0	104	18.6	n.d.	n.d.
10^f^	15:85	18	15:85	18.6	37.8	2.0	147	38.4	−50	51
11^f^	10:90	19	10:90	25.5	49.5	1.9	209	55.0	−52	54
12^f^	5:95	20	5:95	37.1	79.3	2.1	314	97.3	−59	55

^a^ Reaction conditions: 1. **LU/A1**/Sn(Oct)_2_ = 50:1:1, [**LU**] = 1 m in toluene, 100°C, 12 h; 2. Addition of **CL** solution into toluene, 100°C, 3–8 h.

^b^ Ratio of polymer blocks determined by ^1^H NMR.

^c^ Number‐average molar mass (*M*_n_), weight‐average molar mass (*M*_w_), and dispersity (*Đ* = *M*_w_/*M*_n_) determined via gel permeation chromatography (GPC) in DMF with 2.096 g/L LiBr added at 30°C referenced to poly(methylmethacrylate) calibration standards.

^d^ Degree of polymerization (DP) determined by ^1^H NMR and GPC; DP = *M*_n_ /(([**PLU**] × *M*_LU_) + ([**PCL**] × *M*_CL_)).

^e^ Theoretical number‐average molar mass (*M*_n,theo_) determined by ^1^H NMR; *M*_n,theo_ = ([**LU**] × *M*_LU_ × *X*_LU_/[Sn(Oct)_2_]) + ([**CL**] × *M*_CL_ × *X*_CL_/[Sn(Oct)_2_]).

^f^ Copolymerization performed on a gram scale.

Furthermore, the successful synthesis and the different compositions of the **PLU‐b‐PCL** copolymers were analyzed by FT‐IR spectroscopy. As shown in Figure 1C, the intensity of the N─H and C═C valence vibrations and the C─H deformation vibrations from the ring plane of the **PLU** block decreases appropriately with increasing **PCL** ratio and disappears entirely for pure **PCL**. Comparing the carbonyl region from *δ* = 145–180 ppm in the ^13^C NMR spectra of the **PLU‐b‐PCL** copolymer with the **PLU** and **PCL** homopolymers, it can be seen that the chemical shifts of the urethane and ester moieties occur at the same chemical shifts of approximately *δ* = 149 and 174 ppm, respectively (Figures S12, S14, and S16).

As can be seen in Table 1, the composition of the **PLU‐b‐PCL** copolymers is within the range of the **LU**/**CL** monomer ratios applied. The **PLU‐b‐PCL** composition can be finely adjusted during the synthesis and was successfully carried out over a wide range from 65:35 to 5:95 with several gradations. Regardless of the composition and average molecular weight *M*_n_, the dispersity *Đ* for all copolymers is in the range of about 2.0. It is worth mentioning that even by upscaling the ROCOP for material studies from small batches of less than 100 mg to the gram range, both the composition of the **PLU‐b‐PCL** copolymers and their dispersity *Đ* remained almost unchanged (Table 1 entries 7–12). For the polymerizations carried out in both batch sizes, the average molecular weight *M*_n_ is also in the same order of magnitude, demonstrating this method's reproducibility in larger‐scale preparations. However, when a **CL** content of more than 70% is used in the starting material (Table 1 entries 10–12), *M*_n_ deviates increasingly from *M*_n,theo_, indicating transesterification during polymerization caused by chain transfer agents such as **LU**, increased amount of impurities such as water at lower catalyst loadings, or system viscosity, which has already been reported for the ROP of **CL** at elevated temperatures [26, 27].

**FIGURE 2:**
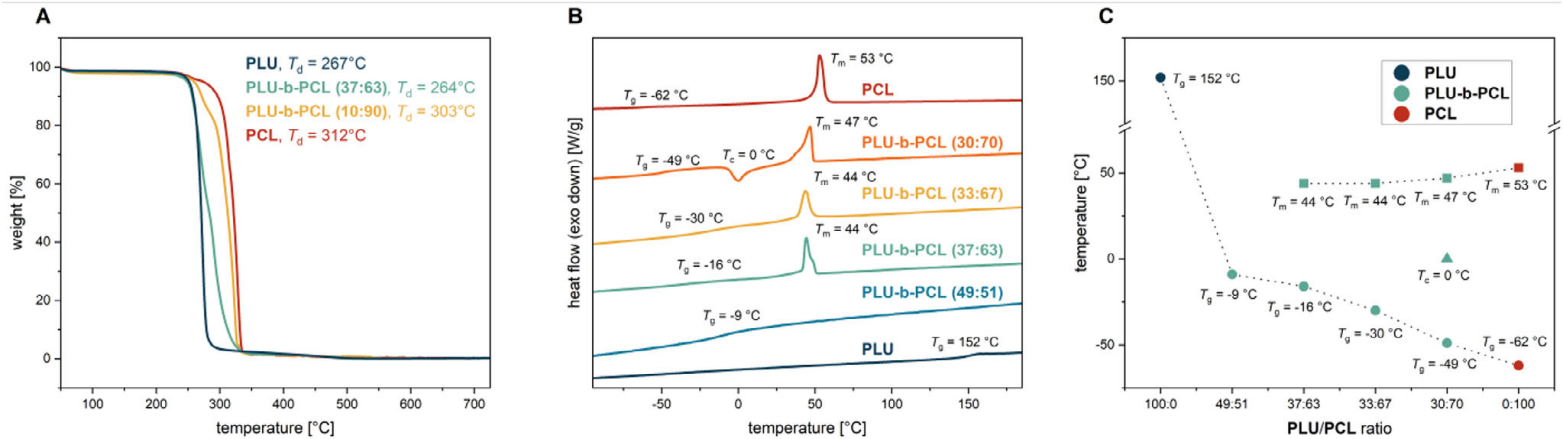
Thermal properties of **PLU** (blue curves), **PLU‐b‐PCL** (light blue, green, yellow, and orange curves for different polymer block compositions), and **PCL** (red curves). (A) TGA thermograms (10 K/min) reveal a *T*_d_ for **PLU‐b‐PCL** in the range between **PLU** and **PCL**. (B) DSC thermograms (5 K/min) of the second heating scan. Before thermal analyses, polymer samples were dried at 80°C for 40 h under vacuum. (C) Plot of *T*_g_, *T*_c_, and *T*_m_ obtained by DSC versus the different **PLU/PCL** ratios of the polymers.

The thermogravimetric analysis (TGA) thermograms of the **PLU‐b‐PCL** copolymers shown in Figure 2A reveal a merged degradation profile of the two polymers, confirming the presence of both monomers. The decomposition temperature *T*_d_ of the **PLU‐b‐PCL** samples (green and yellow curves) is within the range of 267–312°C between the homopolymers **PLU** (blue curve) and **PCL** (red curve) and increases with a higher **PCL** content. The TGA thermograms of the remaining **PLU‐b‐PCL** copolymers are listed in the Supporting Information (see Figure S26).

The differential scanning calorimetry (DSC) thermograms of the polymer samples shown in Figure 2B exhibit different thermal properties depending on their composition. **PLU‐b‐PCL (30:70)** is the only polymer that shows crystallization at *T*_c_ = 0°C in the second heating cycle (orange curve). In contrast, only a glass transition temperature of *T*_g_ = −9°C is measured for **PLU‐b‐PCL (49:51)** (light blue curve), and no further thermal transition is observed. The DSC thermograms of the remaining copolymers with a **PLU**/**PCL** ratio of 15:85 and lower are shown in the Supporting Information (see Figure S27). Repeatedly, the thermal properties of the **PLU‐b‐PCL** copolymers show a tendency to change with increasing **PCL** ratio, affecting the glass transition temperature *T*_g_ and melting temperature *T*_m_. As the plot of the transition temperatures against the composition of the polymers in Figure 2C demonstrates, the glass transition temperature (circles) decreases from *T*_g_ = 152°C for the **PLU** homopolymer (blue) across the copolymers (green) from −9°C for **PLU‐b‐PCL (49:51)** with increasing **PCL** content gradually via *T*_g_ = −49°C for **PLU‐b‐PCL (30:70)** to −62°C for the **PCL** homopolymer (red). Similarly, the melting temperature (squares) of the copolymers from **PLU‐b‐PCL (37:63)** increases from *T*_m_ = 44°C with increasing **PCL** content up to the **PCL** homopolymer with *T*_m_ = 53°C, whereby the temperature range is not as wide as for the glass transition temperature. The DSC measurements indicate that, based on the copolymer composition, the glass transition temperature of **PLU‐b‐PCL** can be adjusted over a wide temperature range of about 50°C.

**FIGURE 3:**
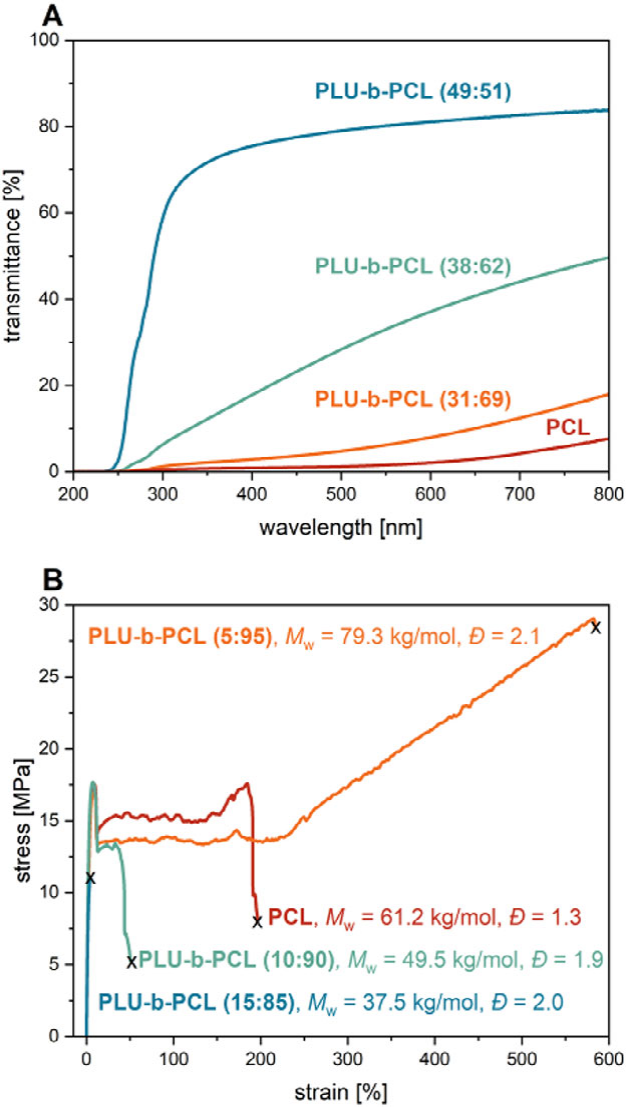
Optical and mechanical properties of **PCL** and various selected **PLU‐b‐PCL** copolymers. (A) UV–vis spectra of 150 µm thick polymer films produced by hot pressing. (B) Stress‐strain curves of various **PLU‐b‐PCL** compositions and **PCL**.

By hot press molding, we prepared films of selected polymers to investigate their optical properties using UV–vis. As shown in Figure 3A, the optical properties of the **PLU‐b‐PCL** copolymers also change with increasing **PCL** content. For example, the transparent film of **PLU‐b‐PCL (49:51)** has a transmission of up to 84% in the range of *λ* = 400–800 nm, whereby the transmission decreases rapidly below *λ* = 400 nm (blue curve). In contrast, the somewhat cloudy film of **PLU‐b‐PCL (38:62)** only exhibits a transmission of around 50% at *λ* = 800 nm, which decreases continuously until *λ* = 250 nm (green curve). Ultimately, the UV–vis spectrum of **PLU‐b‐PCL (31:69)** is strongly resembling that of the opaque **PCL** film, with transmissions below 18% and 8% being measured in the entire range of *λ* = 200–800 nm (orange and red curves, respectively). As demonstrated by the UV–vis measurements of the transparent to opaque polymer films, **PLU‐b‐PCL** has an adjustable transmittance in the range of *λ* = 400–800 nm based on its composition.

In order to investigate the influence of the **PLU‐b‐PCL** composition on the material properties, dog bone‐shaped specimens were produced by hot press molding, and tensile testing was conducted. The **PLU‐b‐PCL** specimens with a high **PLU** content were all very brittle, so the focus for the stress‐strain measurements was set on the copolymers with a high **PCL** content. For instance, the **PLU** homopolymer is so brittle that hot pressing of test specimens is impossible, as they break as soon as they are removed from the mold. The high crystallinity and brittleness of **PLU** can be attributed to the high density of urethane groups in the polymer backbone, the stereoregularity introduced by the monomer **LU**, or a combination of both factors [21]. The representative stress‐strain curves shown in Figure 3B reveal significant effects of even a small proportion of the **PLU** block in the **PLU‐b‐PCL** copolymers compared to the **PCL** homopolymer. It must be mentioned that the hard **PLU** block of all copolymers was kept constant, and therefore, the block length of the soft **PCL** block varies in addition to *M*_w_ (37.8–79.3 kg/mol). For all **PLU‐b‐PCL** copolymers tested, Young's modulus *E*_t_ was in the range of 530 MPa, indicating stiffer **PLU‐b‐PCL** copolymers than **PCL**, whose *E*_t_ = 380 MPa (Table 2). Thus, the specimen of **PLU‐b‐PCL (15:85)** already breaks after an elongation at break *ε* of 4% (light blue curve), and with a reduced **PLU** content of **PLU‐b‐PCL (10:90)** after 52% (green curve). However, most strikingly, elongation at break *ε* of 582% was measured for the **PLU‐b‐PCL (5:95)** samples, which corresponds to an almost threefold elongation compared to pure **PCL** (210%) (orange and red curves, respectively). Thereby, the ultimate tensile strength *σ* was 29.8 MPa, about twice as high as the other polymers. This gives **PLU‐b‐PCL (5:95)** similar material properties to synthetic poly(3‐hydroxybutyrate) (**PHB**) and isotactic polypropylene (***i*‐PP**) [29]. It would be interesting to investigate the influence of longer **PLU** blocks on the mechanical properties of the copolymers; however, due to the low solubility of **PLU** in toluene, we have not yet succeeded in significantly enlarging the hard segment. In summary, **PLU‐b‐PCL** copolymers produced by COROP are stiff, tough, and ductile materials that can be customized by varying the polymer composition.

**TABLE 2:** Averaged tensile testing results.

Polymer	*E*_t_	*σ*	*ε*
	(MPa)	(MPa)	(%)
PLU‐b‐PCL (15:85)	541 ± 96.5	11.0 ± 0.70	3.93 ± 1.10
PLU‐b‐PCL (10:90)	523 ± 75.6	14.9 ± 1.73	52.4 ± 8.53
PLU‐b‐PCL (5:95)	530 ± 80.3	29.8 ± 3.53	582 ± 59.5
PCL	380 ± 51.7	17.4 ± 1.15	210 ± 18.1

## 3 Conclusion

In conclusion, we have introduced the synthesis of polyesterurethanes by utilizing the ring‐opening copolymerization of limonene‐based cyclic carbamates and *ε*‐caprolactone. Key findings demonstrate that this method provides a viable, phosgene‐ and isocyanate‐free alternative to conventional polyurethane production with the ability to fine‐tune the copolymer properties by varying the monomer ratios. The synthesized **PLU‐b‐PCL** block copolymers exhibited tunable properties such as adjustable glass transition temperatures (−9°C to −59°C), high optical transmittance (up to 84%), and customizable mechanical performance. Notably, **PLU‐b‐PCL (5:95)** achieved an elongation at break of 582%, significantly surpassing pure **PCL**. Overall, this work establishes ring‐opening copolymerization as a reliable method for phosgene‐ and isocyanate‐free polyesterurethanes, advancing sustainable polymer synthesis and promoting greener polyurethane production with reduced environmental impact. Future studies intend to broaden the ring‐opening copolymerization to polyester‐ and polyetherurethanes based on limonene, 3‐carene and *α*‐pinene cyclic carbamates and a wider range of lactones and ethers. In addition, attempts are underway to produce BAB block copolymers using organocatalysts to expand the scope of isocyanate‐ and phosgene‐free polyurethane synthesis via ring‐opening copolymerization.

## Author Contributions

The manuscript was written through the contributions of all authors. All authors have given approval to the final version of the manuscript. J. Futter: conceptualization, data curation, formal analysis, visualization, writing—review & editing; H. Pfaadt: visualization, data curation, formal analysis, review & editing; B. Rieger*: funding acquisition, supervision, project administration, resources, writing—review & editing.

## Conflicts of Interest

The authors declare no conflicts of interest.

## Supporting information




**Supporting File**: marc70115‐sup‐0001‐SuppMat.docx.

## Data Availability

The data that support the findings of this study are available in the supplementary material of this article.
