# Supplementary material for: Coordinative Ring‐Opening Copolymerization of Limonene Carbamate and ε‐Caprolactone Toward Phosgene‐ and Isocyanate‐Free Polyesterurethane Block‐Copolymers with Tunable Properties
Source: Macromol Rapid Commun. 2025 Nov 29;47(1):e00817. doi: 10.1002/marc.202500817 (PMC12784182; doi:10.1002/marc.202500817)
Supplement: Supplementary file 1 — Supporting File: marc70115‐sup‐0001‐SuppMat.docx. [file MARC-47-e00817-s001.docx]

*Supporting Information for:*

**Coordinative Ring-Opening Copolymerization of Limonene Carbamate and ε-Caprolactone towards Phosgene- and Isocyanate-Free Polyesterurethane Block-Copolymers with Tunable Properties**

Jonas Futter^a^, Hendrik Pfaad ^a^, and Bernhard Rieger^a,*^

*^a^* WACKER-Chair of Macromolecular Chemistry, Catalysis Research Center, Department of Chemistry, Technical University of Munich, Lichtenbergstr. 4, 85748 Garching (Germany)

Corresponding Author

*E-mail [rieger@tum.de](mailto:rieger@tum.de) (B.R.).

****Table of Contents****

1. General experimental 2
2. Synthesis procedures 4
3. Polymerization procedure 15
4. References 27

**1. GENERAL EXPERIMENTAL**

All reactions and polymerizations with moisture and air-sensitive reactants were carried out in an M*Braun* LabMaster120 glovebox filled with argon 4.6 from *Westfalen* or using standard *Schlenk* techniques. All glassware was heat-dried before use. All chemicals were purchased from *Sigma-Aldrich*, ABCR, or TCI Europe and used without further purification unless otherwise stated. The catalyst Sn(Oct)_2_ was distilled prior to use, and monomer batches of *ε*-caprolactone (**CL**) were dried over CaH_2_ for one day prior to distillation. Dichloromethane, tetrahydrofuran, and toluene were dried using an M*Braun* SPS-800 solvent purification system and stored over a 3 Å molecular sieve.

**Thin layer chromatography (TLC)** was performed with silica pre-coated polyester sheets (*Macherey-Nagel*, 0.20 mm silica 60, F254). The detection of the molecules took place with UV light (*λ* = 254, 365 nm) or aqueous potassium permanganate solution. Column chromatography was conducted with *Merck* silica (230-400 mesh) and the described eluent.

**Nuclear magnetic resonance (NMR)** spectra were recorded on a *Bruker* AV-400HD at 400 MHz (^1^H) and 100 MHz (^13^C) at ambient temperature. The chemical shifts are stated in *δ*-units relative to the residual proton or carbon signal of deuterated chloroform [CHCl_3_: *δ* (^1^H) = 7.26 ppm, *δ* (^13^C) = 77.16 ppm]. Deuterated solvents were purchased from *Sigma-Aldrich* or *Deutero* and dried over 3 Å molecular sieves before use. Spectra interpretation was performed using MestreNova software. The indication of the atoms was done by italicizing the respective fragment of the molecule, and not according to the IUPAC numbering. Spectral data is provided as follows: chemical shift (multiplicity, integration, coupling constant, assignment). The multiplicity of the signals was assigned as follows: bs - broad singlet, s - singlet, d - doublet, t - triplet, q - quartet, m - multiplet or combination thereof. Coupling constants are stated in Hz and given as averaged values from the experimental data.

**Gas-chromatography mass spectrometry (GC-MS)** measurements were performed on an GC-7890B from *Agilent Technologies* equipped with a MSD 59771 mass detector, a 7693 automatic liquid sampler and a G4513A auto injector. Sample separation is done using a HP-5MS UI column (30.0 m length, 0.25 mm diameter, 0.25 μm film) in a temperature range of 60-300 °C followed by mass spectrometry using full scan method in a mass range of 40-500 au. Samples are prepared by dissolving 1 mg/mL in HPLC grade acetonitrile prior to measurement.

**Elemental analyses (EA)** were carried out by the micro analytical laboratory of the faculty of chemistry at the Technical University of Munich (TUM) using a Vario EL from *Elementar*.

**Gel permeation chromatography (GPC)** was used to determined average molecular weights *M_n_*, *M_w_* and dispersities (*Đ*) of the polymers with a sample concentration of 2 mg/mL. Measurements were performed on an *Agilent* PL-GPC 50 with an integrated RI unit, two light scattering detectors (15° and 90°) and a differential pressure viscosimeter with two *Agilent* PolarGel M columns. As eluent *N*,*N*-dimethylformamide (with 2.096 g/L lithium bromide added) at 30 °C was used with a flow rate of 1.0 mL/min and the resulting spectra were referenced to poly(methylmethacrylate) calibration standards. Due to solubility issues GPC measurements of pure **PCL** samples were performed on a PL-GPC50 plus from *Polymer Laboratories* with THF (with 222 mg/L 3,5-di-*tert*-butyl-4-hydroxytoluol as a stabilizing agent) as eluent at 30 °C. Size separation was done using two PolarGel Mixed-C columns by *Agilent Technologies*. Molecular weight and dispersity are measured *via* single RI-detection and reported against polystyrene standards.

**Fourier-transform infrared spectroscopy (FT-IR)** measurements were recorded on a nitrogen-cooled *Bruker* Vertex 70A spectroscope on an attenuated total reflection module.

**Thermogravimetric analysis (TGA)** was performed from 1-2 mg samples on a TGA Q5000 by *TA Instruments*. Samples were heated from room temperature to 700 °C with a heat rate of 10 K/min under argon. Analysis of mass loss and determination of *T*_d,5%_ is done using *TA Analysis* software.

**Differential scanning calorimetry (DSC)** was measured using a DSC Q2000 by *TA Instruments* in exo-down mode. Sample size is about 6-10 mg in non-hermetic aluminum pans in the range of –95 °C to 185 °C. Analysis is performed using *TA Analysis*.

**Stress-Strain measurements** were performed on a Zmart.Pro by *ZwickRoell* with a strain rate of 5 mm/min and analyzed with testXpert II software. Specimens (dog-bone-shaped, 50 mm long, 4.0 mm wide, 0.50 mm thick; parameters were checked prior to stress-strain measurements) were obtained by compression molding of the solvent-free polymer on a *Servitec* Polystat 200 T at temperatures 10 °C higher than each material’s respective melting temperature *T*_m_ in a two-part cycle: First, the polymer was equilibrated with no applied pressure for 4 min, and second, pressurized at 200 bar for 3 min. The specimens were checked regarding a homogeneous distribution. For polymers without a *T*_m_ a temperature 80 °C above the *T*_g_ was used.

**Ultraviolet-visible spectroscopy (UV-Vis)**: Polymer films with a thickness of 150 ± 5 μm were prepared by hot pressing the polymer between two Teflon sheets 10 °C above the respective melting temperature *T*_m_. The transmission (in %) of polymer film samples was measured using a Cary 60 UV-Vis spectrometer by *Agilent Technologies*, using a scan rate of 600 nm/min.

**2. SYNTHESIS PROCEDURES**

*cis*-Limonene epoxide **1** was prepared according to an adapted literature procedure.^[1]^

**Scheme S1.** Selective synthesis of the monomer **LU** starting from (*R*)-limonene. a) (*R*,*R*)-Mn(III) *Jacobsen* catalyst (0.05 eq.), *m*-CPBA (1.80 eq.), and NMO (3.00 eq.) in DCM at –40 °C for 6 hours. b) NH_3_ (5.00 eq., 25% in water) refluxing for 3 days. c) KO*t*-Bu (1.05 eq.) and DMC (1.00 eq.) in toluene at 100 °C for 20 hours. d) KO*t*-Bu (1.05 eq.) refluxing in THF for 16 hours.

***(1R,4R,6S)-1-methyl-4-(prop-1-en-2-yl)-7-oxabicyclo[4.1.0]heptane*** (**1**):

(*R*)-Limonene (22.0 g, 162 mmol, 1.00 eq.) was dissolved in DCM (450 mL, 0.36 M), and (*R*,*R*)-Mn(III) *Jacobsen* catalyst (5.08 g, 7.87 mmol, 0.05 eq.) and NMO (56.8 g, 485 mmol, 3.00 eq.) were added. m-CBPA (50.2 g, 291 mmol, 1.80 eq.) dissolved in DCM (450 mL) was added dropwise at –40 °C over 2 hours. The mixture was stirred at –40 °C for 4 hours, and reaction progress was monitored by GC-MS. A saturated solution of sodium bicarbonate (400 mL) was added until no gas formation could be observed. The organic phase was separated from the aqueous phase and washed with water (3 × 300 mL). The organic phase was dried over sodium sulfate, filtered, and the solvent was carefully removed by rotary evaporation without a vacuum at 50 °C. The crude product was purified via column chromatography using *n*-pentane/diethyl ether (40:1) as eluent, yielding cis-(R)-limonene oxide **1** (8.19 g, 53.8 mmol, 68%) as a yellow liquid.

TLC: R_f_ = 0.23 (silica, *n*-pentane/diethyl ether 50:1) [KMnO_4_]

^1^H NMR (400 MHz, CDCl_3_) *δ* (ppm) = 4.72 (d, 1H, ^2^*J*_HH_ = 1.5 Hz, H‑10), 4.67 (d, 1H, ^2^*J*_HH_ = 1.5 Hz, H‑10), 3.06 (s, 1H, H‑1), 2.17‑2.08 (m, 2H, H‑5, H‑6), 1.87‑1.83 (m, 2H, H‑3), 1.69 (s, 3H, H‑9), 1.68‑1.64 (m, 1H, H‑6), 1.57‑1.50 (m, 1H, H‑4), 1.31 (s, 3H, H‑7), 1.28‑1.15 (m, 1H, H‑4).

^13^C{^1^H} NMR (100 MHz, CDCl_3_) *δ* (ppm) = 149.19 (C‑8), 109.17 (C‑10), 60.69 (C‑1), 57.50 (C‑2), 36.35 (C‑5), 30.87 (C‑6), 28.77 (C‑3), 26.07 (C‑4), 24.44 (C‑7), 21.24 (C‑9).

Elemental analysis: calc. for C_10_H_16_O: C, 78.90; H, 10.59; O, 10.51; found: C, 78.54; H, 10.40.

ESI-MS: m/z = calc. for [C_10_H_16_O]^+^: 152.1201 ([M]^+^); found 152.1203.

GC-MS: t_R_ = 7.362 min, m/z = 152.1 ([M]^+^), 137.1 ([M-CH_3_]^+^).

**
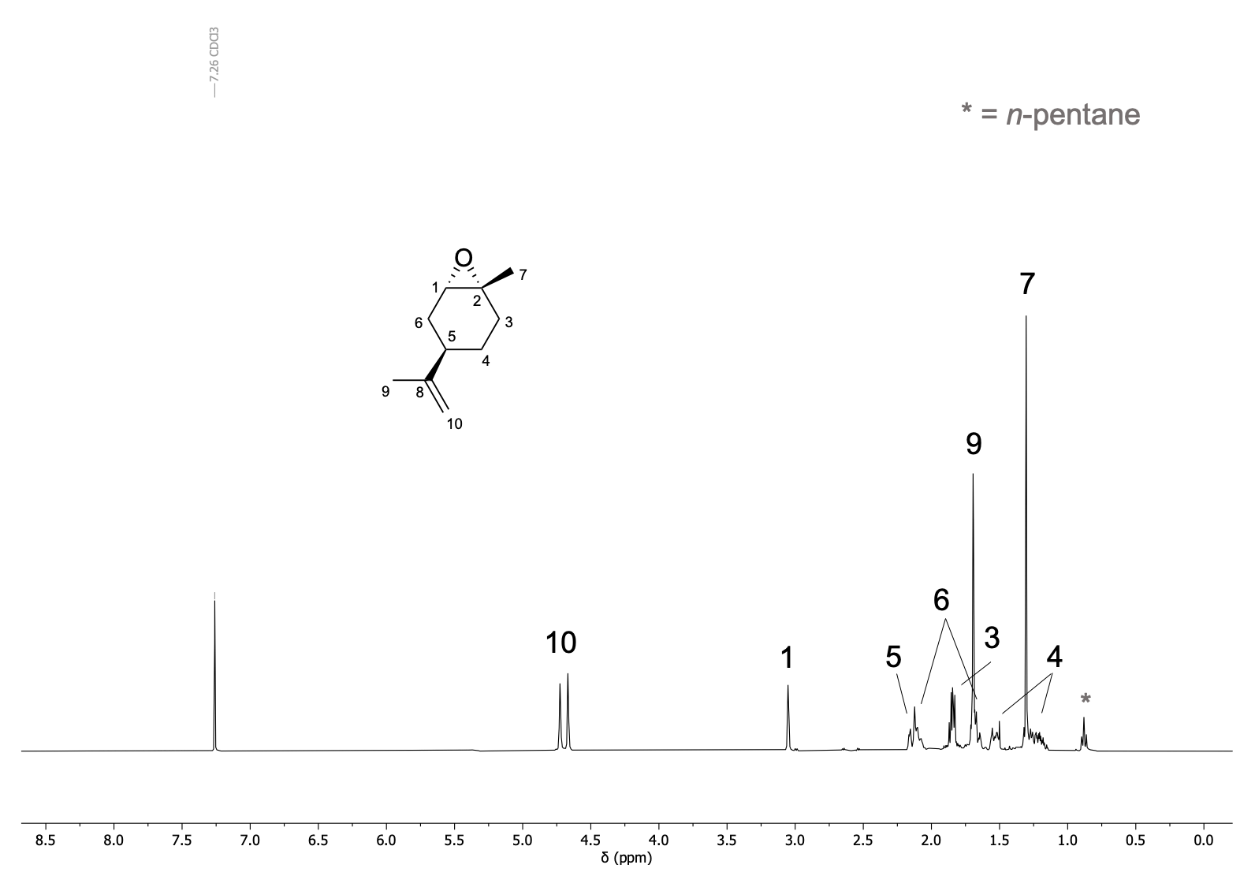
**

**Figure S1.** ^1^H NMR spectrum of *cis*-limonene epoxide **1** in CDCl_3_.

**
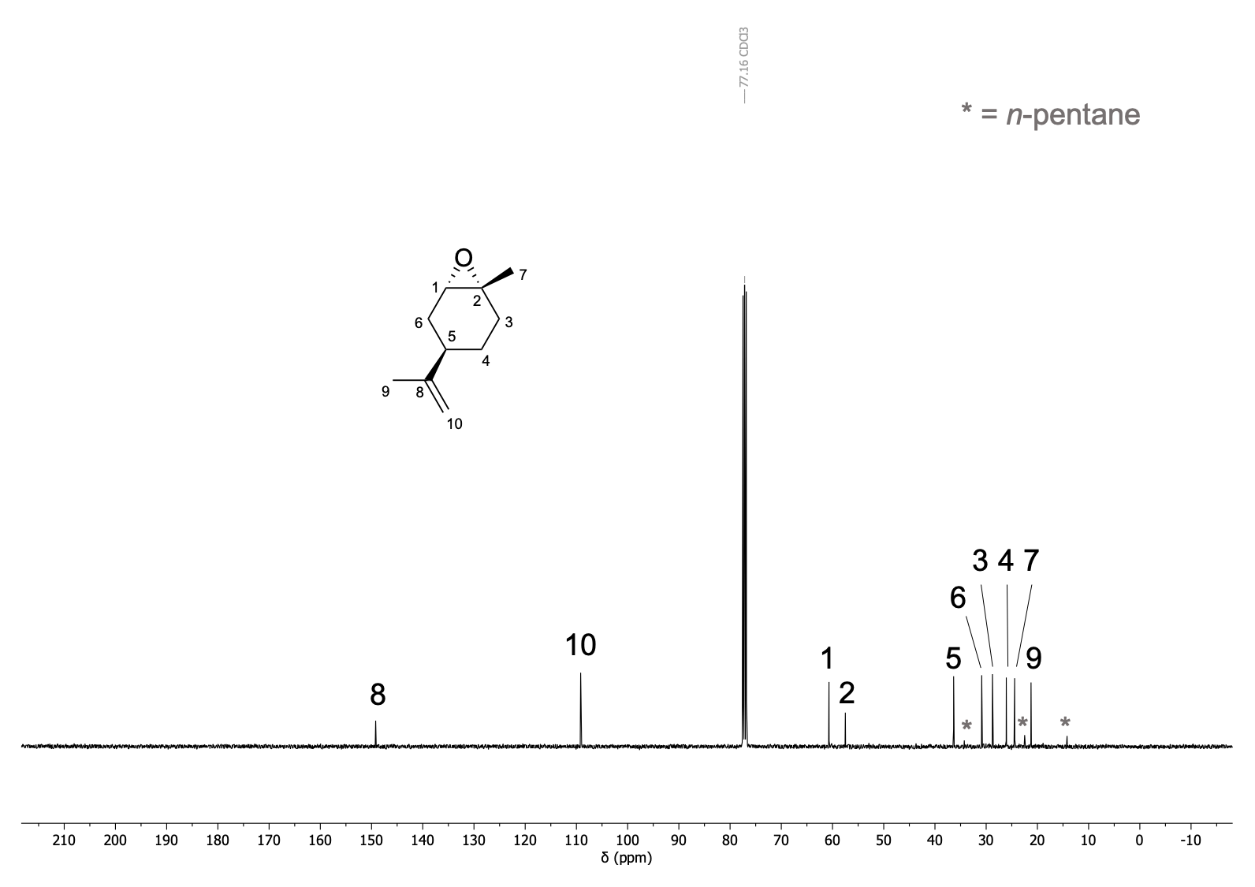
**

**Figure S2.** ^13^C NMR spectrum of *cis*-limonene epoxide **1** in CDCl_3_.

***(1S,2S,5R)-2-amino-2-methyl-5-(prop-1-en-2-yl)cyclohexan-1-ol*** (**2**):

**cis-(R)-limonene oxide **1** (2.5 g, 16.4 mmol, 1.00 eq.) was placed in a pressure tube, and aqueous ammonia (6.20 mL, 25% NH_3_ in water, 82.1 mmol, 5.00 eq.) was added. The pressure tube was sealed, and the reaction was stirred at 100 °C for 3 days. After cooling to room temperature, the water was removed under reduced pressure, resulting in a yellow solid. The crude product was, was dissolved in DCM (50 mL), dried over anhydrous sodium sulfate, filtered, concentrated under reduced pressure, and purified *via* sublimation (80 °C at 10^–5^ mbar), yielding amino alcohol **2** (2.62 g, 15.5 mmol, 94%) as colorless crystals.

TLC: R_f_ = 0.10 (silica, DCM/methanol 9:1) [KMnO_4_]

^1^H NMR (400 MHz, CDCl_3_) *δ* (ppm) = 4.74 (s, 2H, H‑10), 3.55 (t, 1H, ^3^*J*_HH_ = 3.8 Hz, H‑1), 2.28 (td, 1H, ^3^*J*_HH_ = 10.3 Hz, 5.1 Hz, H‑5), 1.92‑1.81 (m, 4H, H‑6, N‑H_2_, O‑H), 1.73 (s, 3H, H‑9), 1.73‑1.63 (m, 2H, H‑3, H‑6), 1.66‑1.56 (m, 1H, H‑4), 1.55‑1.48 (m, 1H, H‑4), 1.44‑1.37 (m, 1H, H‑3), 1.15 (s, 3H, H‑7).

^13^C{^1^H} NMR (100 MHz, CDCl_3_) *δ* (ppm) = 149.14 (C‑8), 109.26 (C‑10), 74.84 (C‑1), 52.01 (C‑2), 37.81 (C‑5), 34.62 (C‑3), 33.75 (C‑6), 26.30 (C‑7), 26.28 (C‑4), 21.49 (C‑9).

Elemental analysis: calc. for C_10_H_19_NO: C, 70.96; H, 11.31; N, 8.28; O, 9.45; found: C, 70.35; H, 11.46; N, 8.05.

ESI-MS: m/z = calc. for [C_10_H_19_NO]^+^: 169.1466 ([M]^+^); found 170.1544 ([M+H]^+^).

GC-MS: t_R_ = 9.350 min, m/z = 169.1 ([M]^+^), 154.1 ([M-CH_3_]^+^).

**
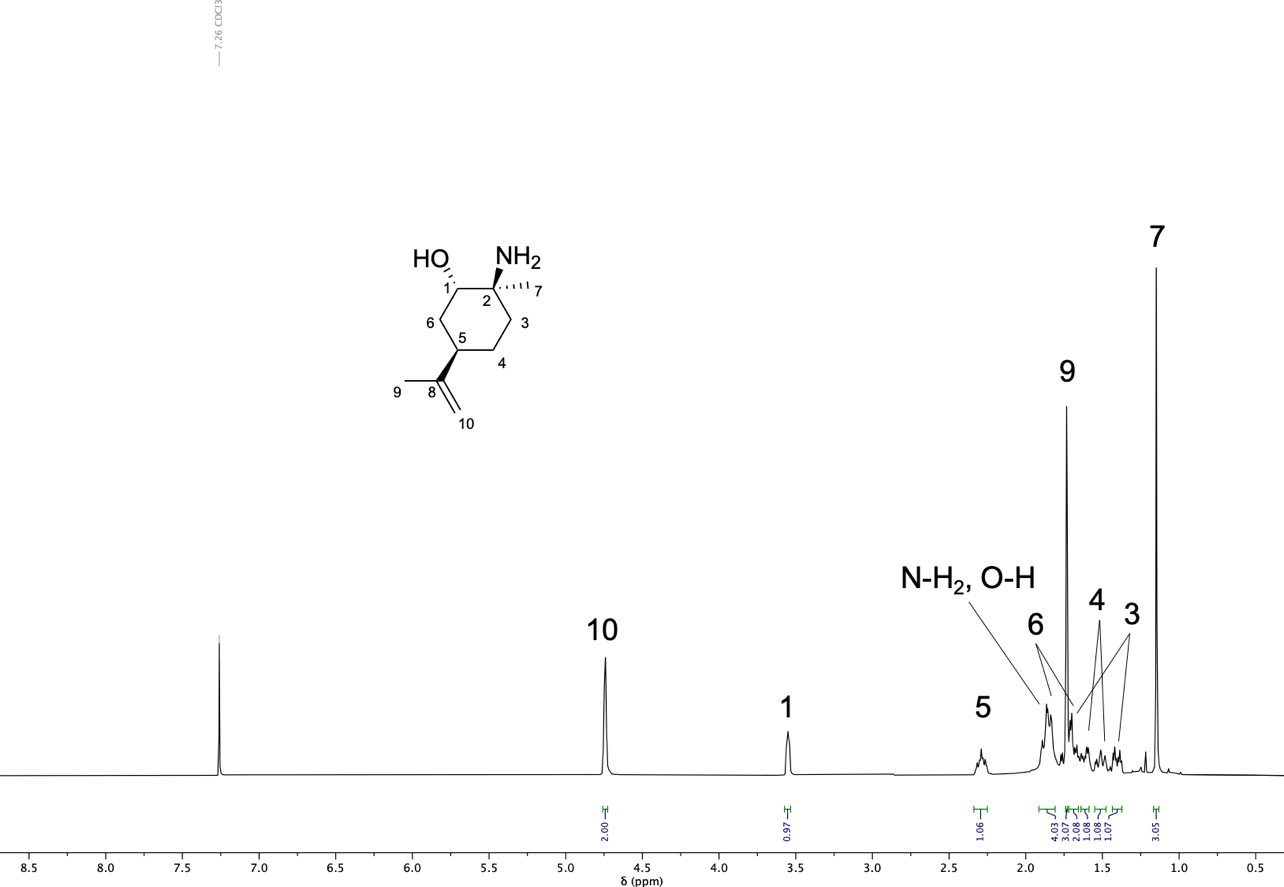
**

**Figure S3.** ^1^H NMR spectrum of amino alcohol **2** in CDCl_3_.

**
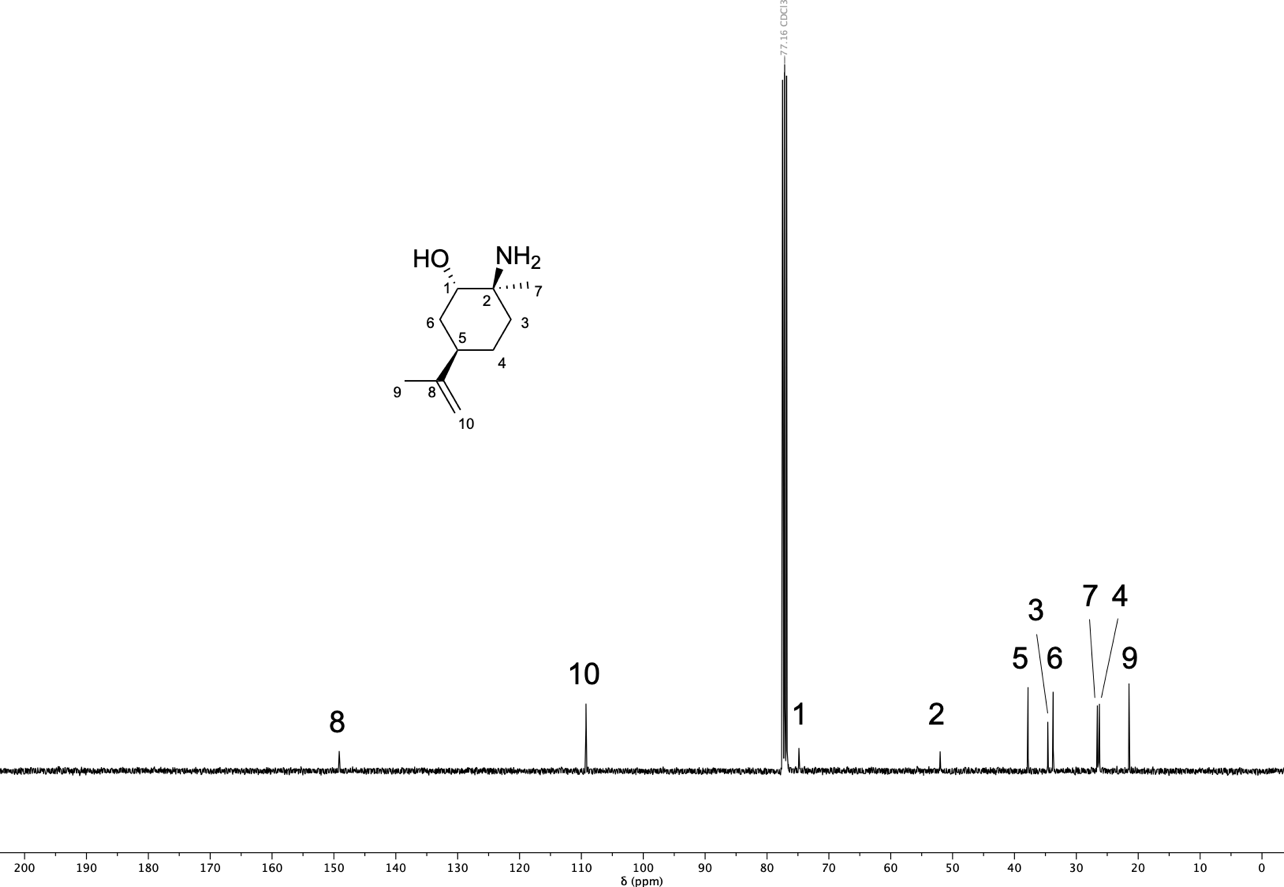
**

**Figure S4.** ^13^C NMR spectrum of amino alcohol **2** in CDCl_3_.

***Methyl ((1S,4R)-2-hydroxy-1-methyl-4-(prop-1-en-2-yl)cyclohexyl)carbamate*** (**3**):

Amino alcohol **2** (5.00 g, 29.5 mmol, 1.00 eq.) was dissolved in toluene (100 mL, 0.30 M) and potassium *tert*-butoxide (3.48 g, 31.0 mmol, 1.05 eq.) was slowly added. After stirring for 30 minutes dimethyl carbonate (2.66 g, 29.5 mmol, 1.00 eq.) was added and the mixture was heated to 100 °C for 20 hours. Toluene was removed under reduced pressure and water (30 mL) was added. The aqueous solution was extracted with DCM (3 × 30 mL). The combined organic layers were dried over anhydrous sodium sulfate, filtered and evaporated under vacuum. The crude product was purified *via* flash column chromatography on silica gel using diethyl ether/*n*-pentane (3:2) as eluent to give ethyl ((1*S*,4*R*)-2-hydroxy-1-methyl-4-(prop-1-en-2-yl)cyclohexyl) carbamate (**3**) (6.36 g, 28.0 mmol, 95%)

TLC: R_f_ = 0.40 (silica, diethyl ether/*n*-pentane 3:2) [KMnO_4_]

^1^H NMR (400 MHz, CDCl_3_) *δ* (ppm) = 4.74 (d, ^2^*J*_HH_ = 11.5 Hz, 2H, H‑11), 4.59 (s, 1H, N‑H), 4.21 (s, 1H, H‑1), 3.62 (s, 3H, H‑12), 2.48 (s, 1H, O‑H), 2.39‑2.27 (m, 1H, H‑6), 1.83‑1.77 (m, 1H, H‑7), 1.76‑1.74 (m, 1H, H‑4), 1.72 (s, 3H, H‑10), 1.71‑1.68 (m, 1H, H‑7), 1.67‑1.64 (m, 1H, H‑4), 1.62‑1.58 (m, 1H, H‑5), 1.48‑1.42 (m, 1H, H‑5), 1.39 (s, 3H, H‑8).

^13^C{^1^H} NMR (100 MHz, CDCl_3_) *δ* (ppm) = 155.93 (C‑2), 148.80 (C‑9), 109.40 (C‑11), 70.49 (C‑1), 55.46 (C‑3), 51.92 (C‑12), 37.23 (C‑6), 33.62 (C‑7), 32.66 (C‑4), 25.72 (C‑5), 21.98 (C‑8), 21.51 (C‑10).

Elemental analysis: calc. for C_12_H_21_NO_3_: C, 63.41; H, 9.31; N, 6.16; O, 21.12; found: C, 63.46; H, 9.32; N, 6.21.

ESI-MS: m/z = calc. for [C_12_H_21_NO_3_]^+^: 227.1521 ([M]^+^); found 227.1525.

GC-MS: t_R_ = 12.408 min, m/z = 227.1 ([M]^+^), 209.1 ([M-H_2_O]^+^).

**
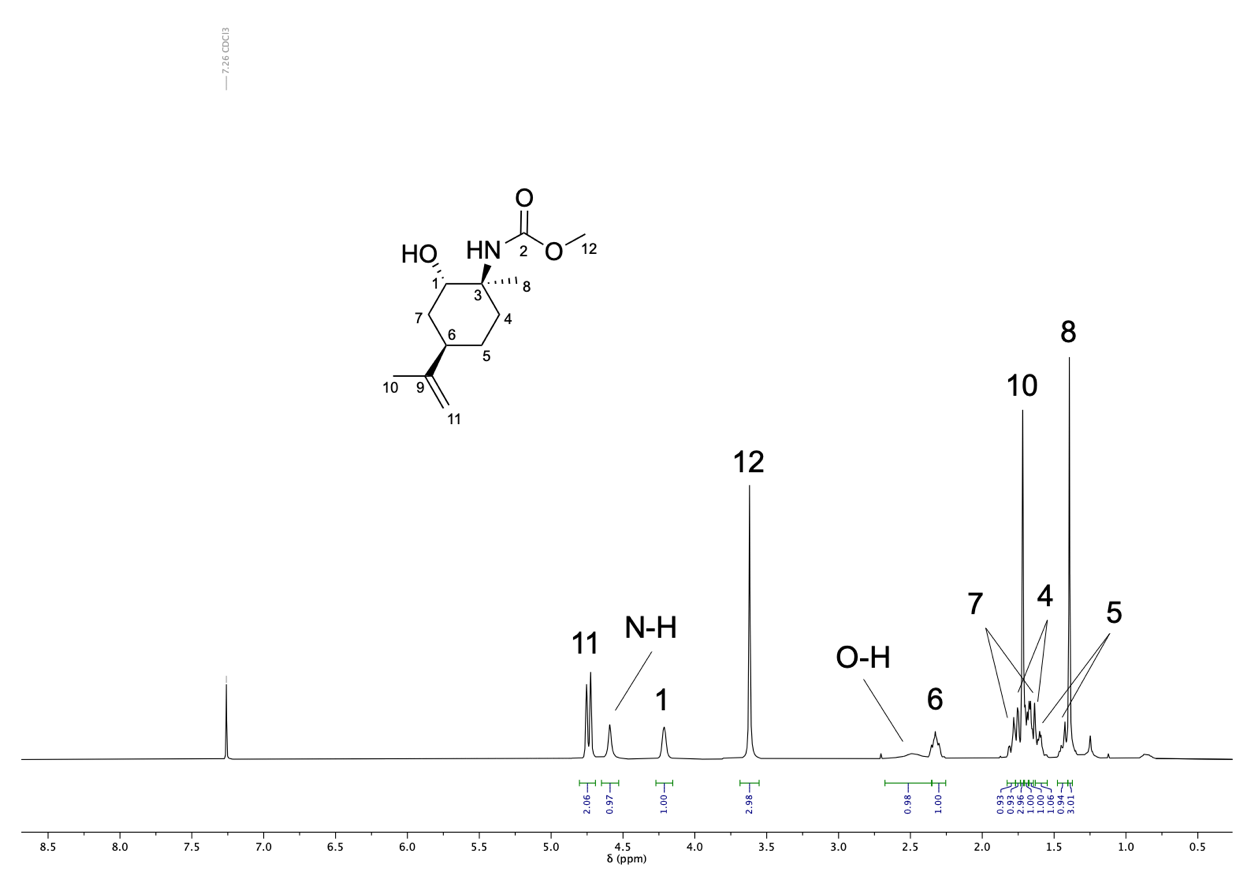
**

**Figure S5.** ^1^H NMR spectrum of carbamate **3** in CDCl_3_.


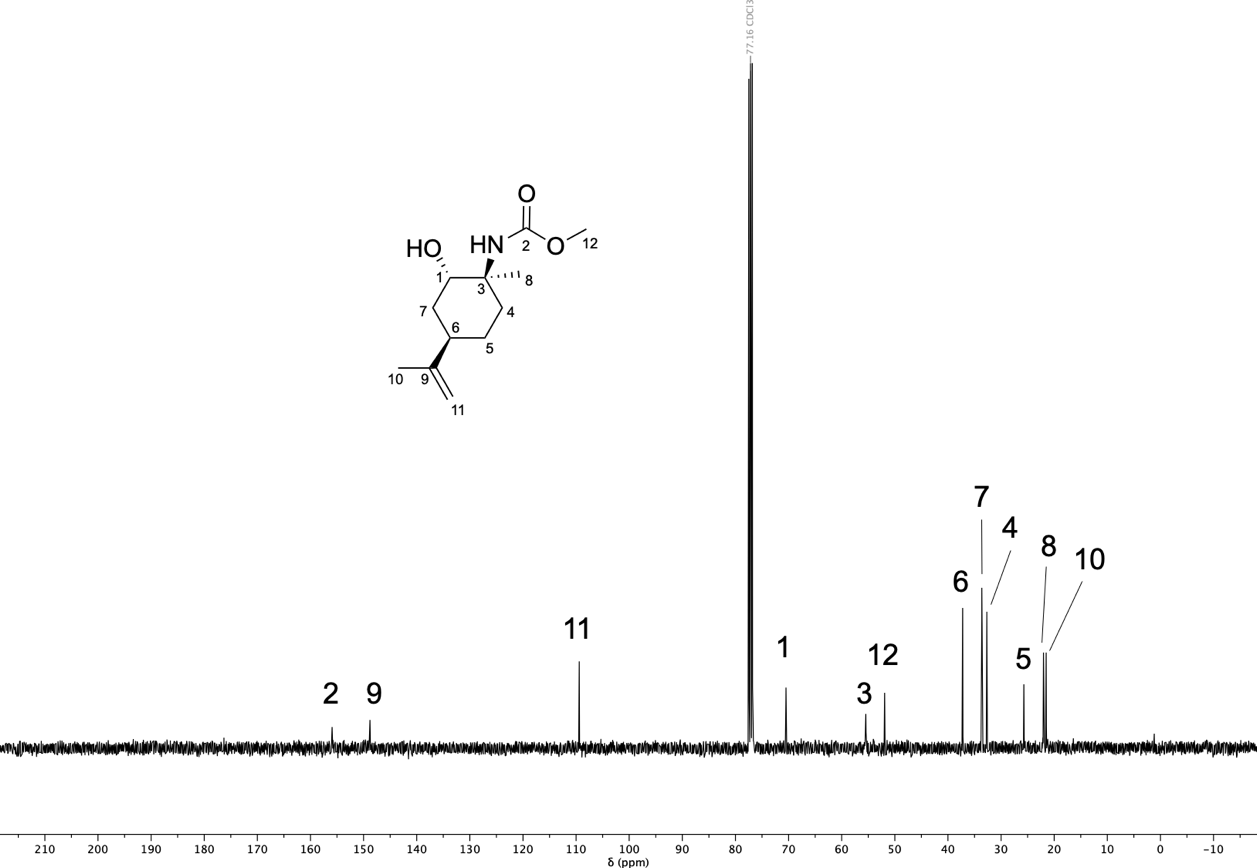


**Figure S6.** ^13^C NMR spectrum of carbamate **3** in CDCl_3_.

***(3aS,6R,7aS)-3a-methyl-6-(prop-1-en-2-yl)hexahydrobenzo[d]oxazol-2(3H)-one*** (**LU**):

A solution of hydroxyl carbamate **3** (5.00 g, 22.0 mmol, 1.00 eq.) in THF (110 mL, 0.20 M) was cooled to 0 °C, and potassium *tert*-butoxide (2.84 g, 25.3 mmol, 1.15 eq.) was added. After stirring for 1 hour, the mixture was refluxed for 16 hours. THF was removed by rotary evaporation, and water (40 mL) was added. The aqueous solution was extracted with DCM (3 × 40 mL). The combined organic layers were dried over anhydrous sodium sulfate, filtered, and evaporated under vacuum. The crude product was purified *via* flash column chromatography on silica gel using diethyl ether/*n*-pentane (3:2) as eluent. The resulting crude product was sublimed at 10^-5^ mbar and 80 °C to get pure (3a*S*,6*R*,7a*S*)-3a-methyl-6-(prop-1-en-2-yl)hexahydrobenzo[*d*]oxazol-2(3*H*)-one (**LU**) (3.57 g, 18.3 mmol, 83%) as a colorless solid.

TLC: R_f_ = 0.56 (silica, diethyl ether) [KMnO_4_]

^1^H NMR (400 MHz, CDCl_3_) *δ* (ppm) = 5.14 (bs, 1H, N‑H), 4.95 (q, 1H, ^2^*J*_HH_ = 1.5 Hz, ^4^*J*_HH_ = 1.3 Hz, H‑11), 4.87 (d, 1H, ^2^*J*_HH_ = 1.5 Hz, H‑11), 4.12 (dd, 1H, ^3^*J*_HH_ = 13.2 Hz, 3.3 Hz, H‑1), 2.57 (t, 1H, ^3^*J*_HH_ = 6.5 Hz, H‑6), 2.20 (ddq, 1H, ^3^*J*_HH_ = 13.2 Hz, 3.3 Hz, 1.5 Hz, H‑7), 2.07‑1.95 (m, 1H, H‑5), 1.94‑1.88 (m, 1H, H‑4), 1.87‑1.82 (m, 1H, H‑7), 1.78 (s, 3H, H‑10), 1.77‑1.74 (m, 1H, H‑4), 1.74‑1.71 (m, 1H, H‑5), 1.25 (s, 3H, H‑8).

^13^C{^1^H} NMR (100 MHz, CDCl_3_) *δ* (ppm) = 160.55 (C‑2), 146.39 (C‑9), 111.69 (C‑11), 81.31 (C‑1), 60.47 (C‑3), 38.93 (C‑6), 32.55 (C‑4), 25.18 (C‑7), 24.46 (C‑5), 22.82 (C‑10), 18.42 (C‑8).

Elemental analysis: calc. for C_11_H_17_NO_2_: C, 67.66; H, 8.78; N, 7.17; O, 16.39; found: C, 67.66; H, 8.90; N, 7.09.

ESI-MS: m/z = calc. for [C_11_H_17_NO_2_]^+^: 195.1259 ([M]^+^); found 195.1259.

GC-MS: t_R_ = 13.473 min, m/z = 195.1 ([M]^+^), 180.1 ([M-CH_3_]^+^).

**
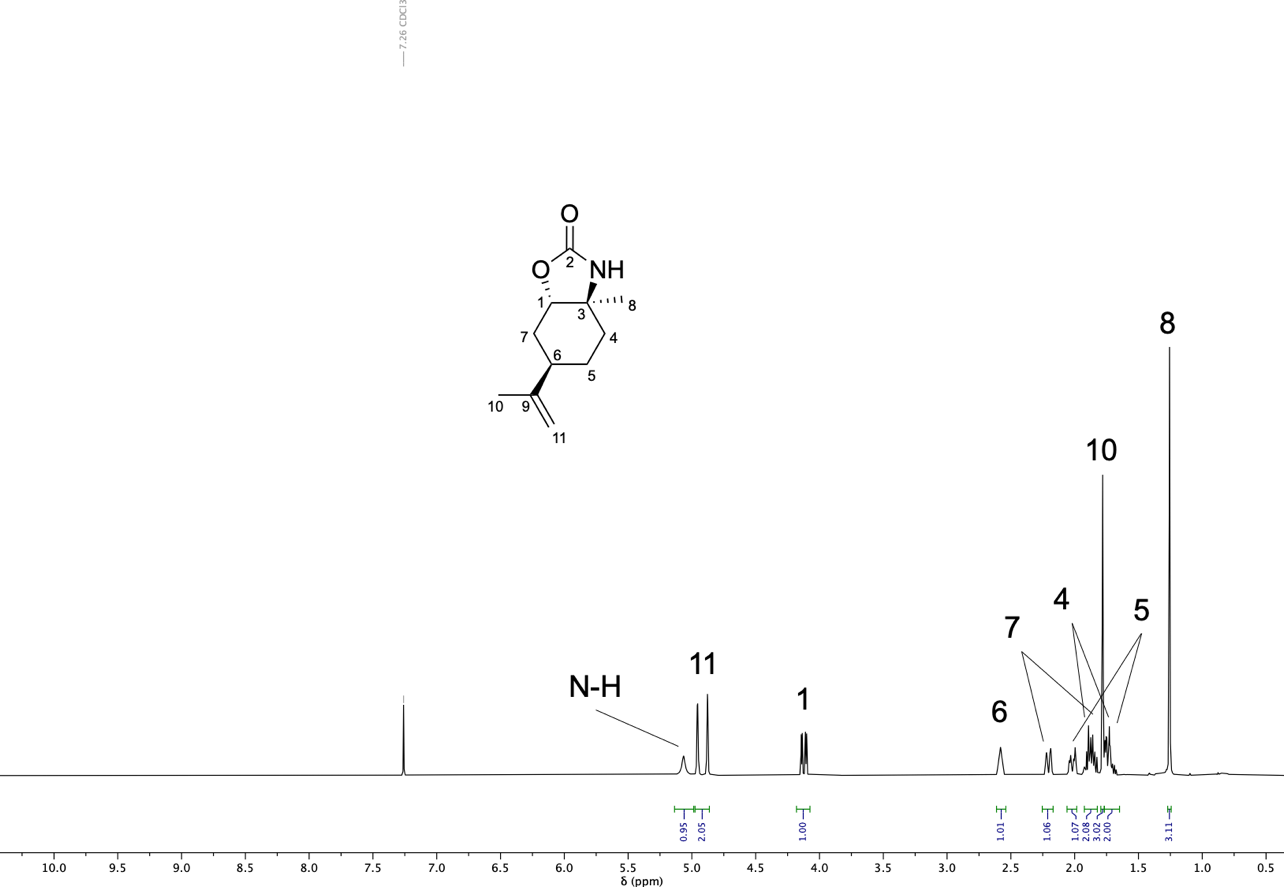
**

**Figure S7.** ^1^H NMR spectrum of monomer **LU** in CDCl_3_.


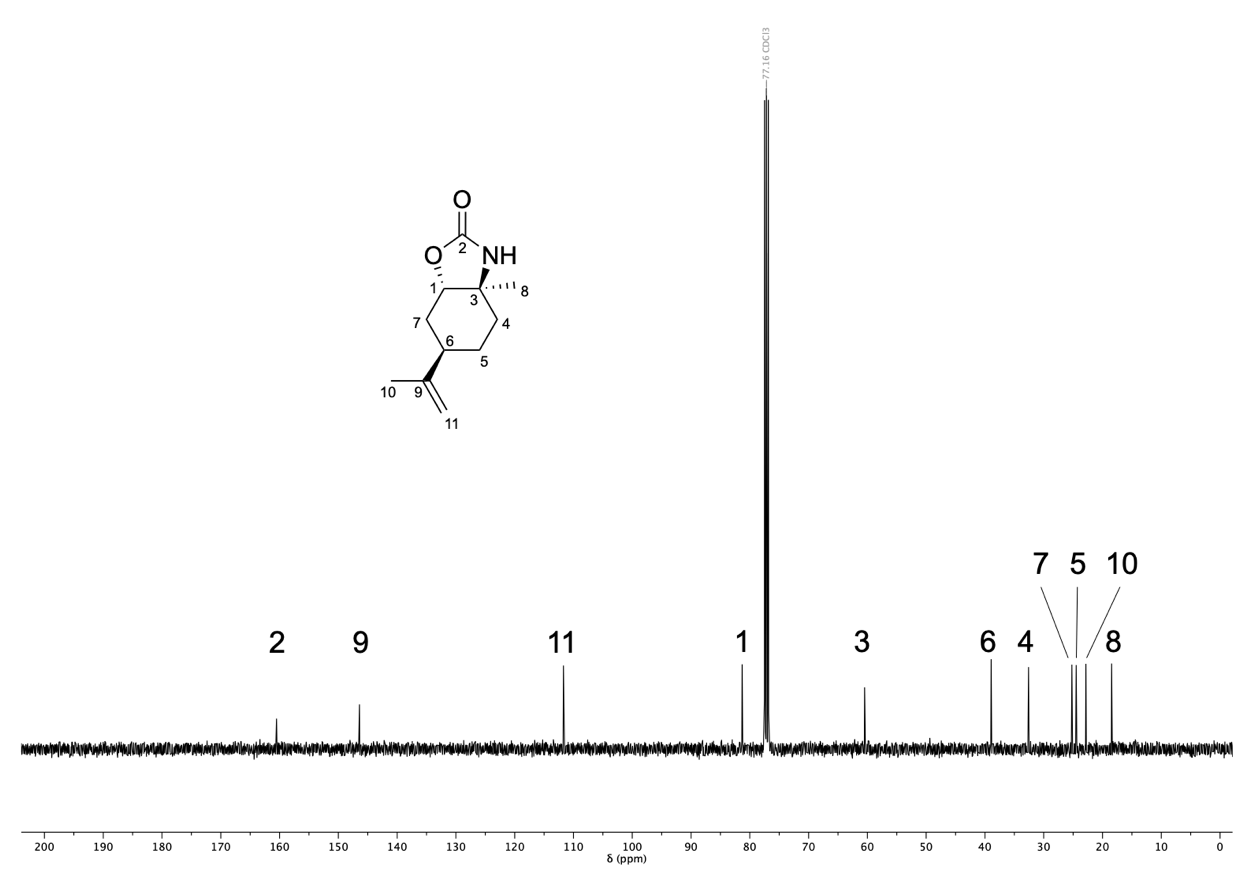


**Figure S8.** ^13^C NMR spectrum of monomer **LU** in CDCl_3_.

*(3aS,6R,7aS)-3-benzoyl-3a-methyl-6-(prop-1-en-2-yl)hexahydrobenzo[d]oxazol-2(3H)-one* (A1): *n*-Butyl lithium (2.50 M in *n*-hexane, 360 μL, 901 μmol, 1.10 eq.) was added to a stirred solution of (3a*S*,6*R*,7a*S*)-3a-methyl-6-(prop-1-en-2-yl)hexahydro-benzo[*d*]oxazol-2(3*H*)-one (LU) (160 mg, 819 μmol, 1.00 eq.) in THF (3 mL, 0.27 M) at –78 °C. After 30 minutes benzoyl chloride (138 mg, 983 μmol, 1.20 eq.) was slowly added. The mixture was stirred for 30 minutes and then slowly heated to room temperature. After 16 hours THF was removed by rotary evaporation and water (5 mL) was added. The aqueous solution was extracted with DCM (3 × 5 mL). The combined organic layers were washed with brine (5 mL) dried over anhydrous sodium sulfate, filtered and evaporated under vacuum. The crude product was purified *via* flash column chromatography on silica gel using *n*‑pentane/ethyl acetate (4:1) as eluent. Recrystallization in *n*‑hexane/ethyl acetate (1:1) gave pure (3a*S*,6*S*,7a*S*)-3-benzoyl-3a-methyl-6-(prop-1-en-2-yl)hexahydrobenzo[*d*]oxazol-2(3*H*)-one (A1) (202 mg, 675 μmol, 82%) as a colorless solid.

TLC: R_f_ = 0.56 (silica, *n*-pentane/ethyl acetate 4:1) [UV]

^1^H NMR (400 MHz, CDCl_3_) *δ* (ppm) = 7.68‑7.65 (m, 2H, H‑14, H‑18), 7.56 (ddt, 1H, ^3^*J*_HH_ = 8.5 Hz, 7.0 Hz, ^4^*J*_HH_ = 1.5 Hz, H‑16), 7.44 (dd, 2H, ^3^*J*_HH_ = 8.5 Hz, 7.0 Hz, H‑15, H‑17), 5.04 (q, 1H, ^2^*J*_HH_ = 1.4 Hz, H‑11), 4.95 (s, 1H, H‑11), 4.26 (dd, 1H, ^3^*J*_HH_ = 13.2 Hz, 3.5 Hz, H‑1), 2.66 (t, 1H, ^3^*J*_HH_ = 6.1 Hz, H‑6), 2.58‑2.48 (m, 1H, H‑4), 2.30 (ddt, 1H, ^3^*J*_HH_ = 13.2 Hz, 3.5 Hz, H‑7), 2.13 (dt, 1H, ^3^*J*_HH_ = 14.5 Hz, 2.1 Hz, H‑5), 2.01 (dd, 1H, ^3^*J*_HH_ = 13.2 Hz, 6.1 Hz, H‑7), 1.98‑1.93 (m, 1H, H‑4), 1.92‑1.87 (m, 1H, H‑5), 1.85 (s, 3H, H‑10), 1.57 (s, 3H, H‑8).

^13^C{^1^H} NMR (100 MHz, CDCl_3_) *δ* (ppm) = 170.27 (C‑12), 154.58 (C‑2), 146.05 (C‑9), 133.90 (C‑13), 132.58 (C‑16), 129.13 (C‑14, C‑18), 128.12 (C‑15, C‑17), 111.97 (C‑11), 80.71 (C‑1), 65.63 (C‑3), 38.53 (C‑6), 30.95 (C‑4), 24.66 (C‑7), 24.07 (C‑5), 22.79 (C‑10), 14.69 (C‑8).

Elemental analysis: calc. for C_18_H_21_NO_3_: C, 72.22; H, 7.07; N, 4.68; O, 16.03; found: C, 72.34; H, 6.98; N, 4.72.

ESI-MS: m/z = calc. for [C_18_H_21_NO_3_]^+^: 299.1599 ([M+H]^+^); found 300.1592.

GC-MS: t_R_ = 17.278 min, m/z = 299.2 ([M]^+^).


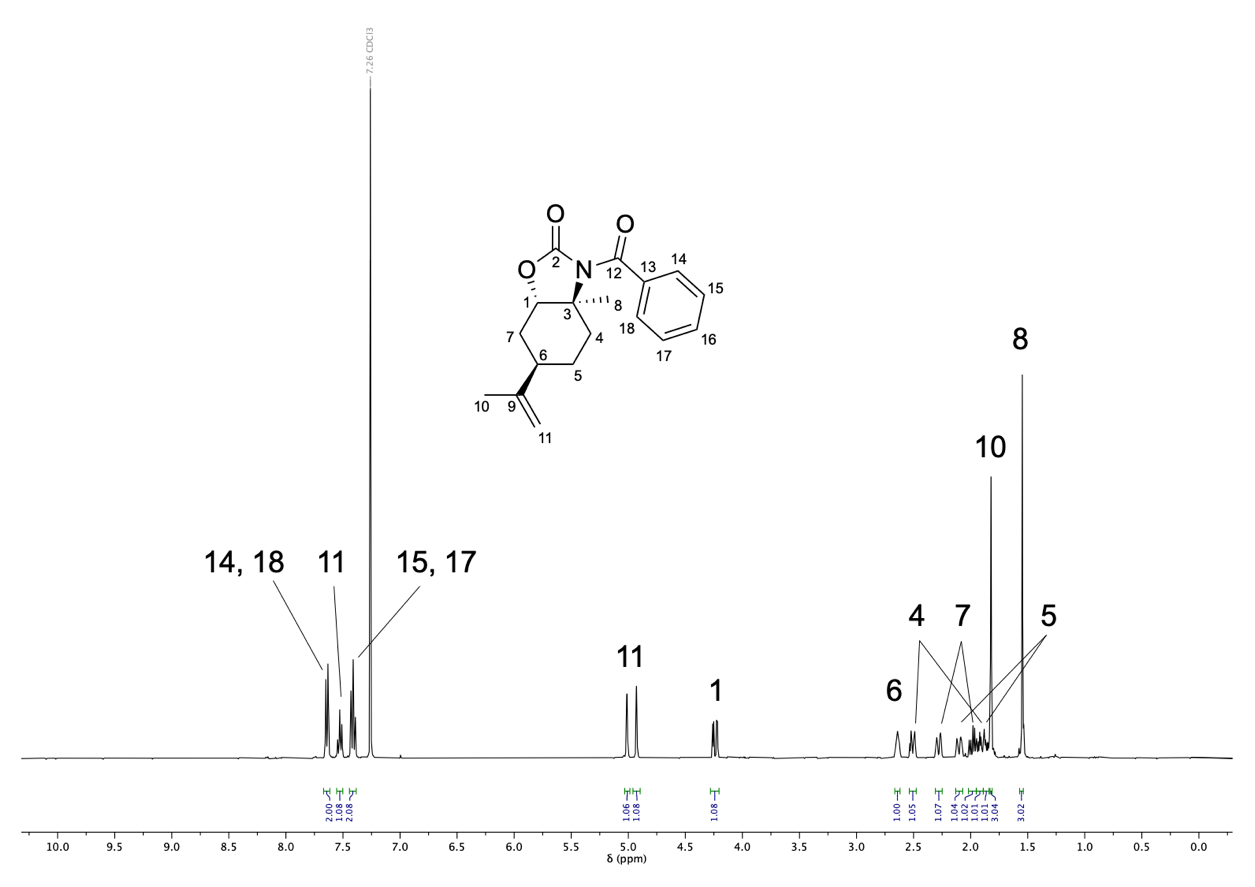


**Figure S9.** ^1^H NMR spectrum of activator **A1** in CDCl_3_.


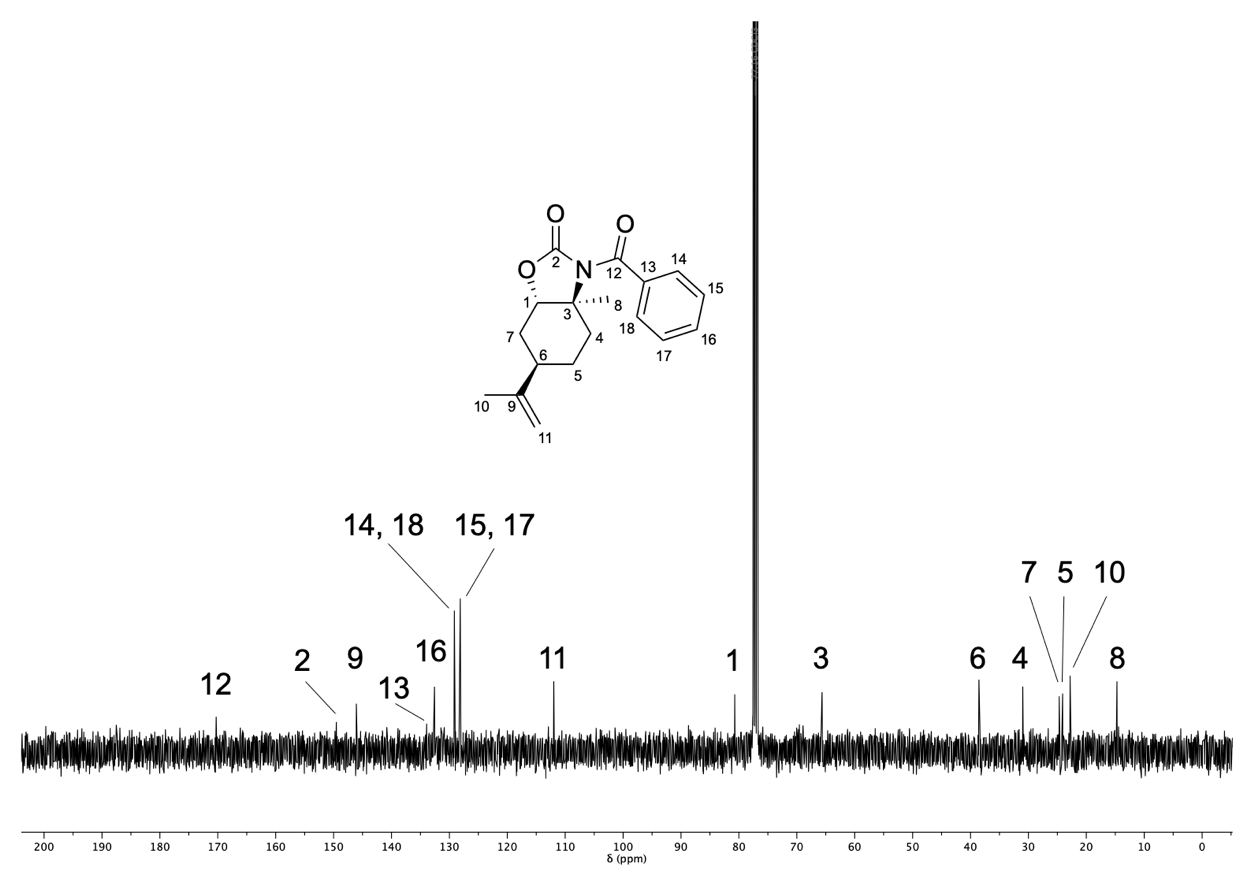


**Figure S10.** ^13^C NMR spectrum of activator **A1** in CDCl_3_.

**3. Polymerization Procedures**

**Polymerization procedure for carbamate monomer LU:**

**All polymerizations were carried out in a glove box under argon atmosphere. At room temperature LU (50.0 mg, 256 µmol, 1.00 eq.) was placed in a crimp vial equipped with a stirring bar and stock solutions of activator A1 and Sn(Oct)_2_ were added to reach the desired equivalents and a concentration of 1 M monomer in toluene (256 µL). The vial was closed and placed in a 100 °C preheated aluminum block.** Polymerizations were performed for 12 hours and quenched by addition of wet CDCl_3_. Aliquots were taken for conversion determination using ^1^H NMR analysis. The polymers were precipitated twice from THF with *n*-pentane, separated *via* centrifugation and dried under vacuum at 80 °C for 48 hours. For material studies, the polymerization procedure was carried out on a gram scale.

^1^H NMR (400 MHz, CDCl_3_) *δ* (ppm) = 5.23 (bs, 1H, N‑H), 4.71 (d, 2H, ^2^*J*_HH_ = 14.1 Hz, H‑11), 4.58 (s, 1H, H‑1), 2.21 (s, 1H, H‑6), 1.97 (s, 1H, H‑5), 1.88‑1.74 (m, 2H, H‑4, H‑5), 1.69 (s, 3H, H‑10), 1.67‑1.53 (m, 2H, H‑4, H‑7), 1.43‑1.25 (m, 4H, H‑7, H‑8).

^13^C{^1^H} NMR (100 MHz, CDCl_3_) *δ* (ppm) = 148.72 (C‑2), 143.67 (C‑9), 109.30 (C‑11), 72.21 (C‑1), 54.37 (C‑3), 38.01 (C‑6), 31.18 (C‑4), 25.91 (C‑7), 22.77 (C‑5), 21.22 (C‑8, C‑10).


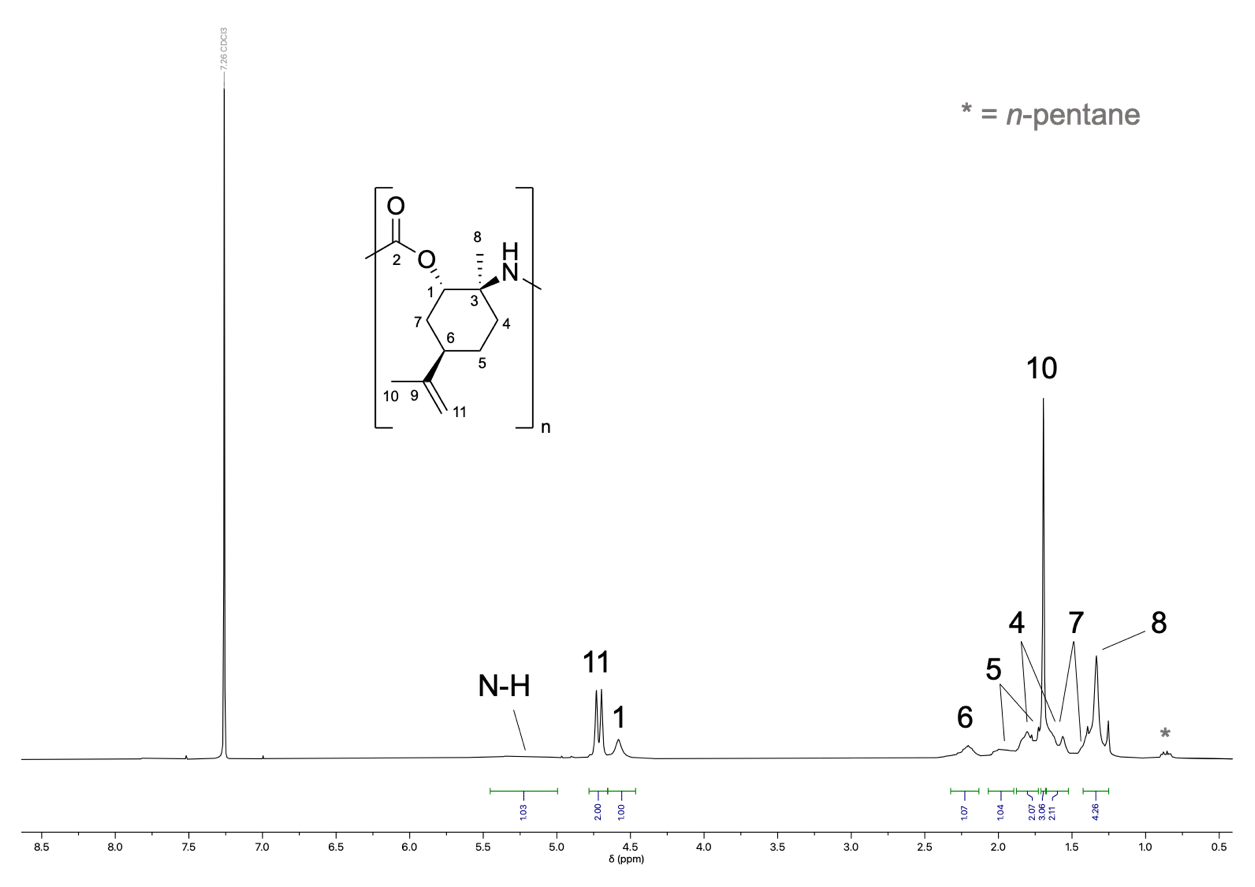


**Figure S11.** ^1^H NMR spectrum of polymer **PLU** in CDCl_3_.


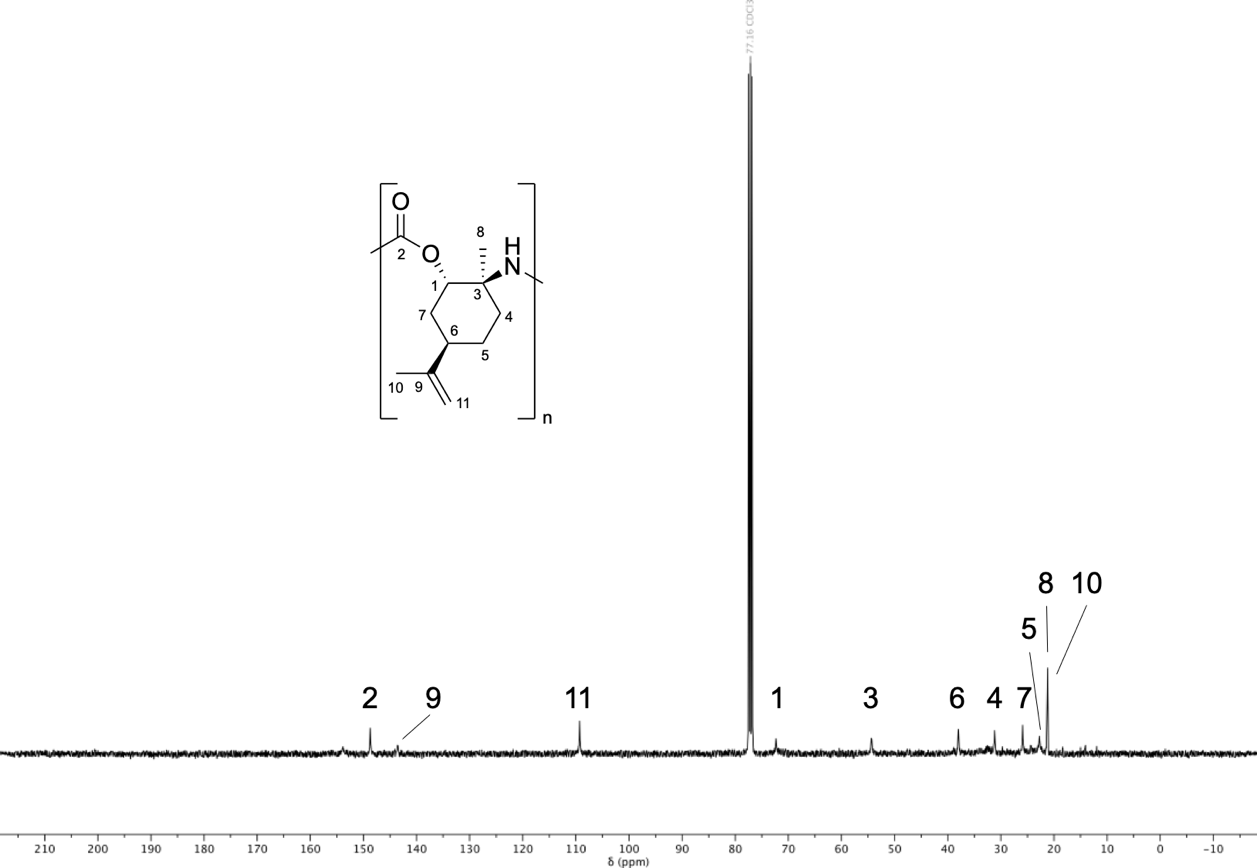


**Figure S12.** ^13^C NMR spectrum of polymer **PLU** in CDCl_3_.

**Polymerization procedure for lactone monomer CL:**

All polymerizations were carried out in a glove box under an argon atmosphere. At room temperature **CL** (50.0 mg, 438 µmol, 1.00 eq.) **was placed in a crimp vial equipped with a stirring bar**. Stock solutions of cyclohexylamine and Sn(Oct)_2_ were added to reach the desired equivalents and a concentration of 1 M monomer in toluene (438 µL). The vial was closed and placed in a 100 °C preheated aluminum block. Polymerizations were performed for a certain time and quenched by addition of wet CDCl_3_. Aliquots were taken for conversion determination using ^1^H NMR analysis. The polymers were precipitated twice from THF with *n*-pentane, separated *via* centrifugation and dried under vacuum at 80 °C for 48 hours. For material studies, the polymerization procedure was carried out on a gram scale.

^1^H NMR (400 MHz, CDCl_3_) *δ* (ppm) = 4.05 (t, 2H, ^3^*J*_HH_ = 6.8 Hz, H‑6), 2.29 (t, 2H, ^3^*J*_HH_ = 7.5 Hz, H‑2), 1.70‑1.57 (m, 4H, H‑3, H‑5), 1.44‑1.31 (m, 2H, H‑4).

**^13^C{^1^H} NMR** (100 MHz, CDCl_3_) *δ* (ppm) = 173.66 (C‑1), 64.26 (C‑6), 34.24 (C‑2), 28.47 (C‑5), 25.66 (C‑4), 24.70 (C‑3).


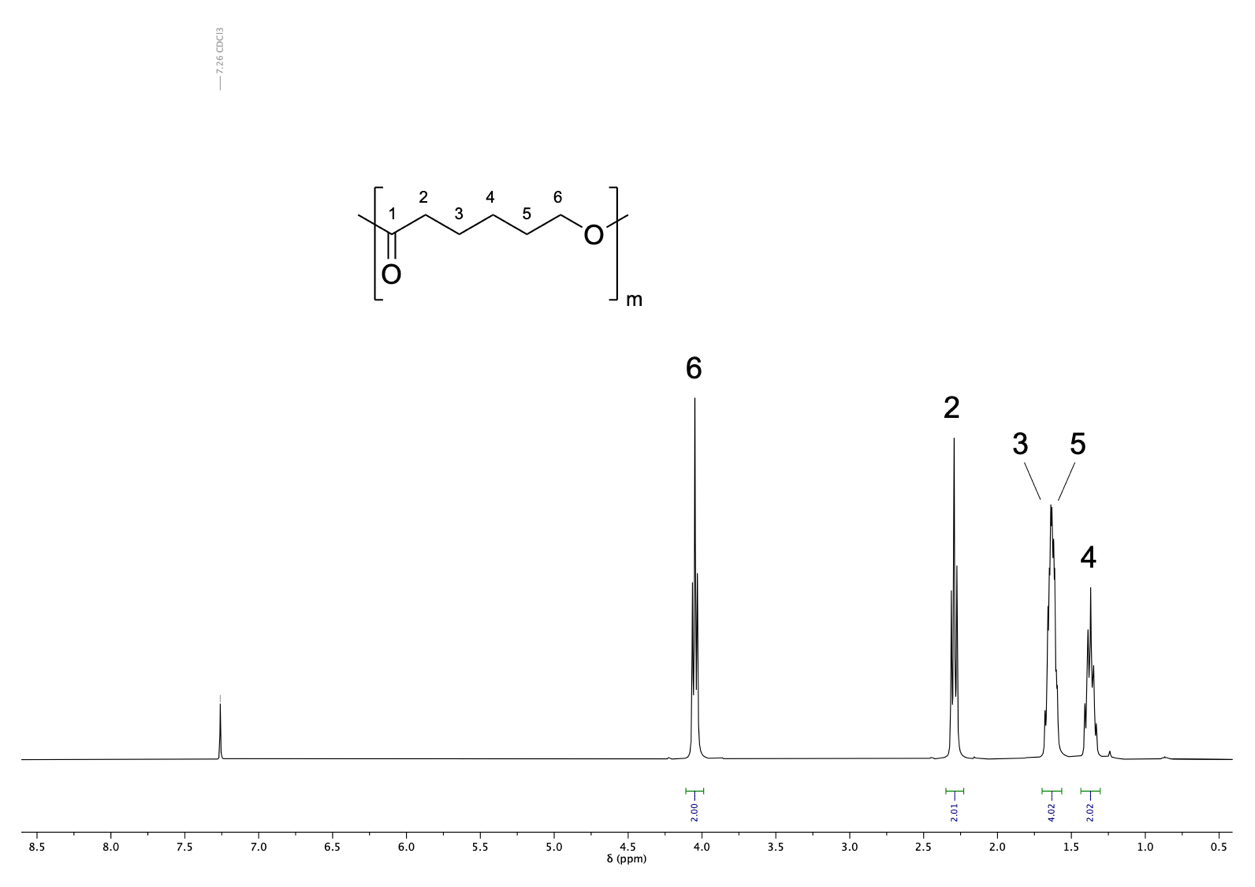


**Figure S13.** ^1^H NMR spectrum of polymer **PCL** in CDCl_3_.


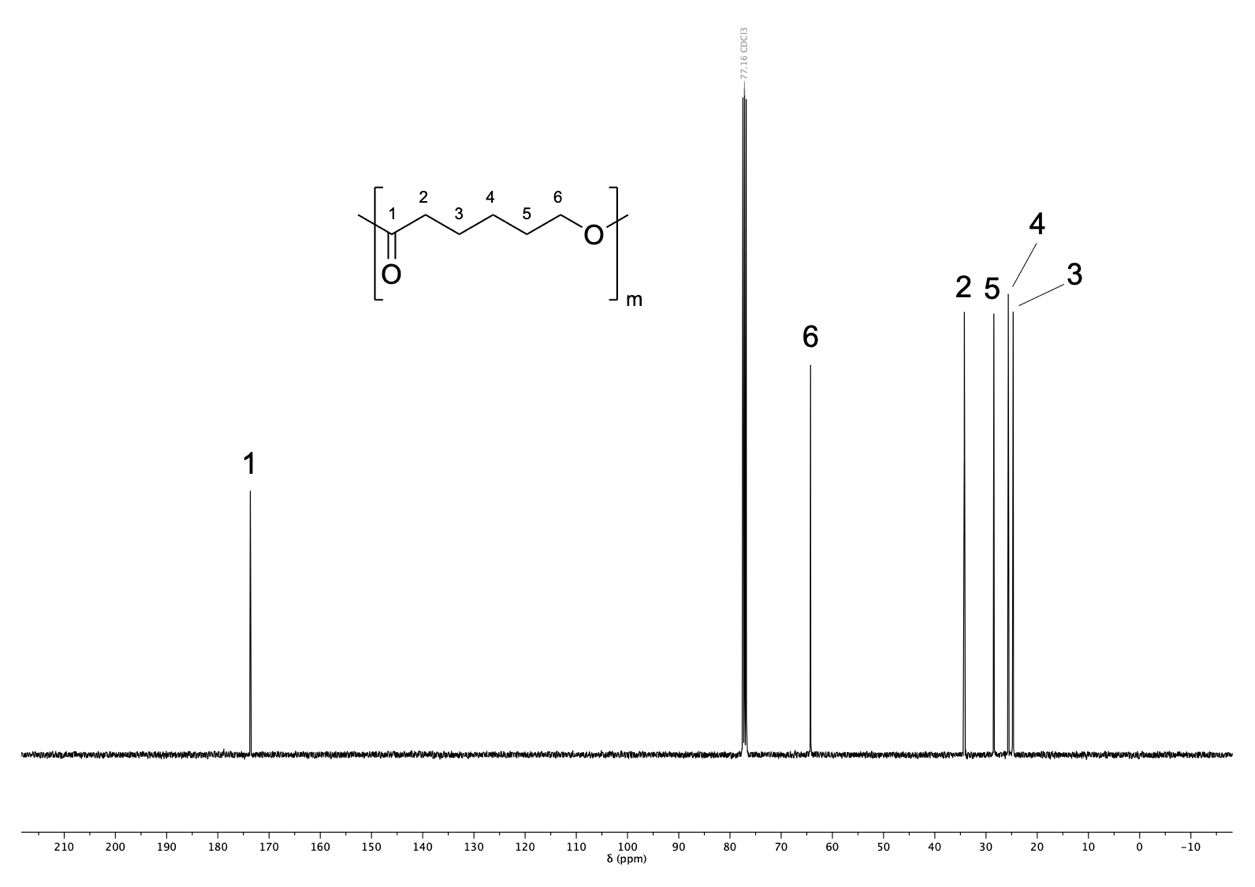


**Figure S14.** ^13^C NMR spectrum of polymer **PCL** in CDCl_3_.

**Copolymerization procedure for carbamate monomer LU and lactone monomer CL:**

**All polymerizations were carried out in a glove box under argon atmosphere. At room temperature LU (50.0 mg, 256 µmol, 1.00 eq.) was placed in a crimp vial equipped with a stirring bar**. Stock solutions of activator **A1** and Sn(Oct)_2_ were added to reach the desired equivalents of 2 mol% each and a concentration of 1 M monomer in toluene (256 µL). The vial was closed and placed in a 100 °C preheated aluminum block and stirred for 12 hours. After polymerization of the **PLU** block, the reaction vial was opened, an aliquot was taken for conversion determination using ^1^H NMR analysis and the desired equivalents of **CL** dissolved in toluene (1 M) were added. The vial was closed and placed back in the aluminum block. Polymerization of the **PCL** block was carried out at 100 °C for a certain time (see Table 1) and quenched by adding wet CDCl_3_. Aliquots were taken for conversion determination using ^1^H NMR analysis. The polymers were precipitated twice from THF with *n*-pentane, separated *via* centrifugation and dried under vacuum at 80 °C for 48 hours. For material studies, the polymerization procedure was carried out on a gram scale.

^1^H NMR (400 MHz, CDCl_3_) *δ* (ppm) = 5.32 (bs, N‑H), 4.71 (d, ^2^*J*_HH_ = 12.9 Hz, H‑11), 4.59 (s, H‑1), 4.06 (t, ^3^*J*_HH_ = 6.7 Hz, H‑17), 2.30 (t, ^3^*J*_HH_ = 7.5 Hz, H‑13), 2.19 (s, H‑6), 1.88‑1.76 (m, H‑4, H‑5), 1.73‑1.60 (m, H‑4, H‑5, H‑7, H‑14, H‑16), 1.46‑1.28 (m, H‑7, H‑8, H‑15).

^13^C{^1^H} NMR (100 MHz, CDCl_3_) *δ* (ppm) = 173.69 (C‑12), 148.75 (C‑2),144.02 (C‑9), 109.33 (C‑11), 72.05 (C‑1), 64.27 (C‑17), 53.73 (C‑3), 37.86 (C‑6), 34.25 (C‑13), 30.79 (C‑4), 28.47 (C‑16), 25.65 (C‑15), 24.70 (C‑14). 24.41 (C‑7), 22.68 (C‑5), 21.22 (C‑8, C‑10).


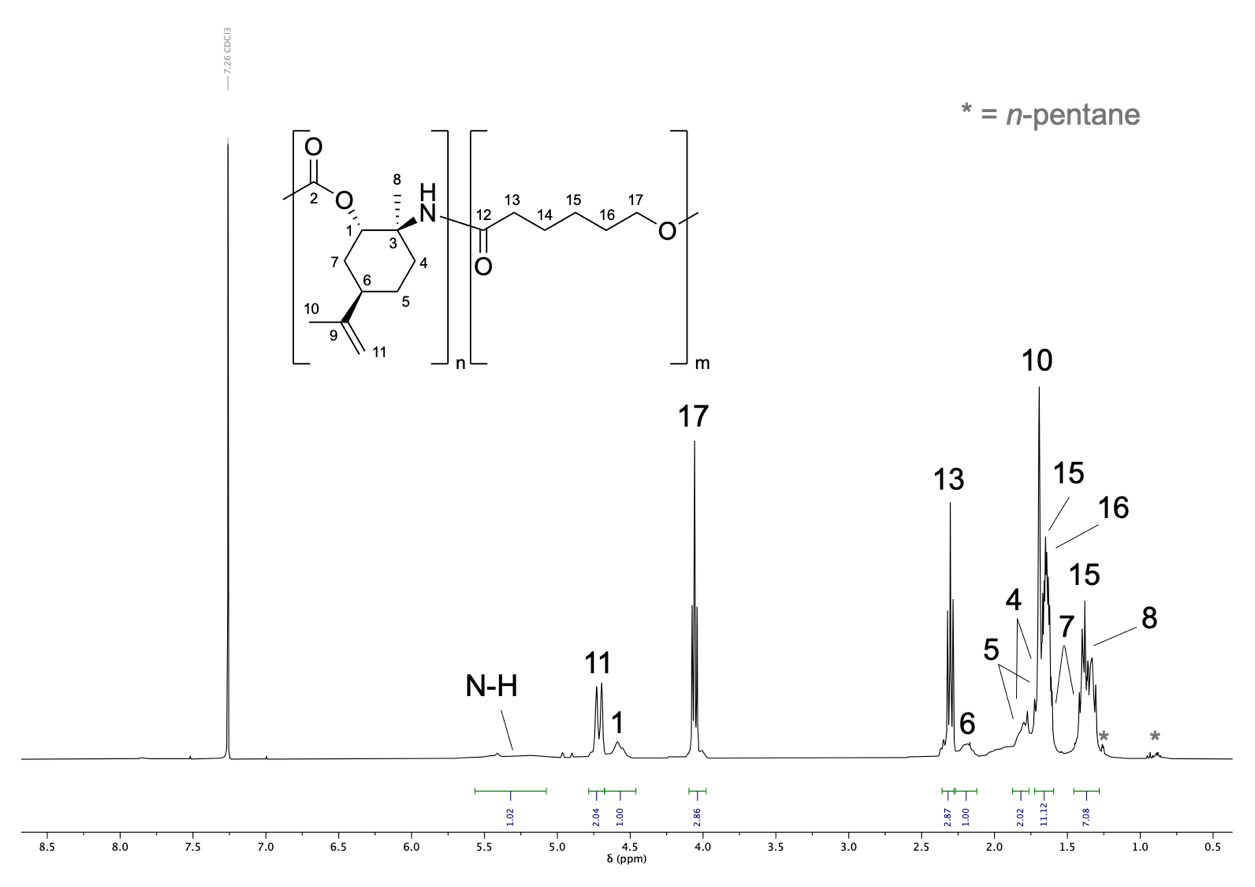


**Figure S15.** Representative ^1^H NMR spectrum of polymer **PLU-b-PCL (42:58)** in CDCl_3_. The proton signal integrals of the **PLU** (1-11) and **PCL** (12-17) blocks vary depending on the **PLU-b-PCL** composition. The copolymer composition was determined by comparing the vinyl (11) and *α*-protons to the ester unit (17) at *δ* = 4.71 ppm and 4.06 ppm for **PLU** and **PCL**, respectively. For the exemplary spectrum for **PLU-b-PCL (42:58)** (Table 1 Entry 3): Proportion **PLU** = I**_PLU_**_vinyl_ / (I**_PLU_**_vinyl_ + I**_PCL_**_ester_) = 2.04 / (2.04 + 2.86) = 0.42; Proportion **PCL** = I**_PCL_**_ester_ / (I**_PLU_**_vinyl_ + I**_PCL_**_ester_) = 2.86 / (2.04 + 2.86) = 0.58.


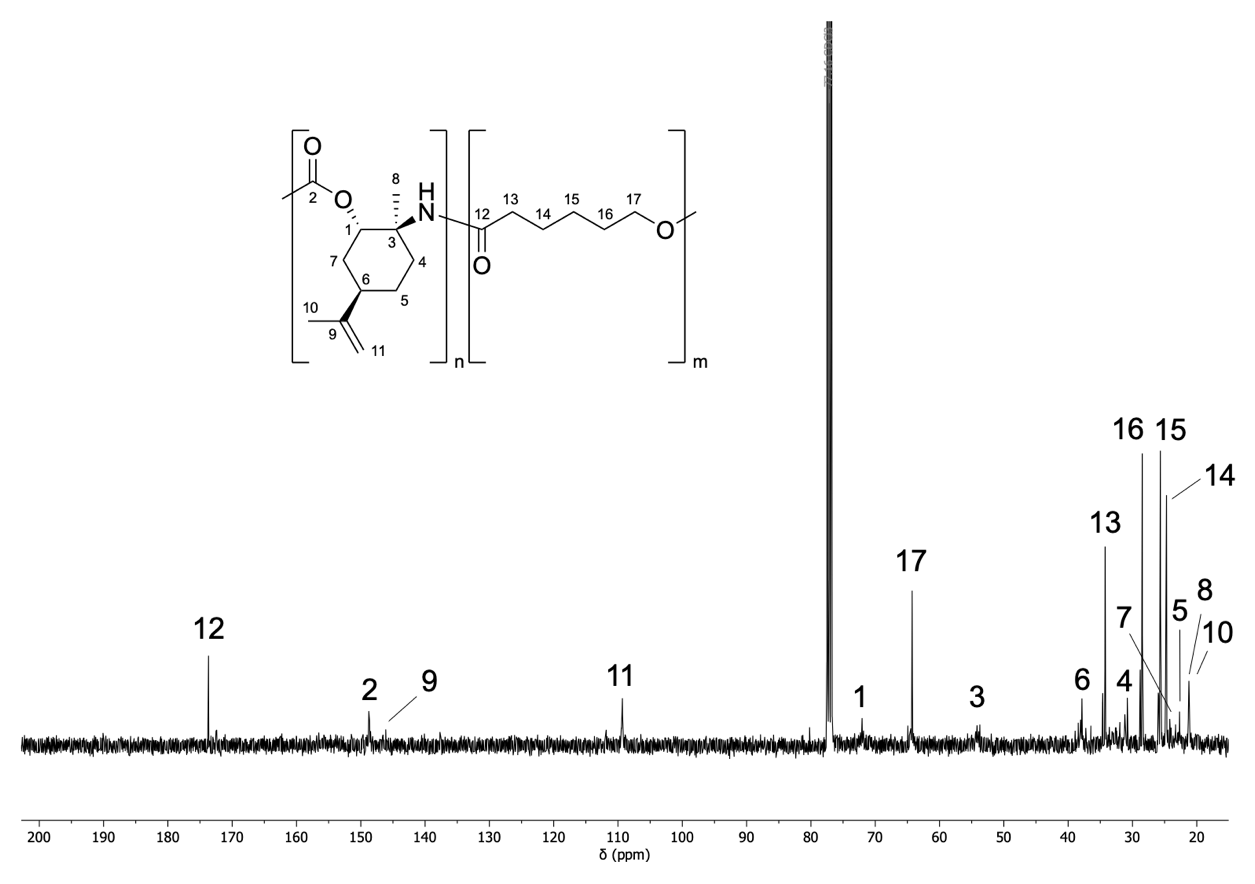


**Figure S16.** ^13^C NMR spectrum of polymer **PLU-b-PCL** in CDCl_3_. According to the chemical shifts in the carbonyl region, the hard block solely contains urethane linkages, as no signals for urea or carbonate moieties were observed. The signal intensity of the **PLU** and **PCL** blocks differs due to relaxation and NOE effects, as observed before for the rigid and soft blocks of polyesterurethanes.^[2]^

**Figure S17.** Kinetic study for the copolymerization of **LU** (blue curve) and **CL** (red curve). Reaction conditions: **LU**/**CL**/**A1**/Sn(Oct)_2_ = 50:50:1:1, 1 M in toluene-*d*_8_, 100 °C.

**Figure S18.** DOSY spectrum of **PLU-b-PCL (49:51)** (Table 1 entry 7).

**Figure S19.** DOSY spectrum of **PLU-b-PCL (38:62)** (Table 1 entry 8).

**Figure S20.** DOSY spectrum of **PLU-b-PCL (31:69)** (Table 1 entry 9).

**Figure S21.** DOSY spectrum of **PLU-b-PCL (15:85)** (Table 1 entry 10).

**Figure S22.** DOSY spectrum of **PLU-b-PCL (10:90)** (Table 1 entry 11).

**Figure S23.** DOSY spectrum of **PLU-b-PCL (5:95)** (Table 1 entry 12).

**Figure S24.** DOSY spectrum of a **PLU**/**PCL** blend **(48:52)**.

**Figure S25.** Representative DOSY spectrum with a non-uniform diffusion coefficient from attempts to produce statistical copolymers. Oligomers with molar masses of less than 2.0 kg/mol were obtained for varying monomer compositions.

**Figure S26.** TGA thermograms (10 °K/min) of **PLU-b-PCL (49:51)** (dark blue curve), **PLU-b-PCL (38:62)** (light blue curve), **PLU-b-PCL (33:67)** (green curve), **PLU-b-PCL (31:69)** (yellow curve), **PLU-b-PCL (15:85)** (orange curve), and **PLU-b-PCL (5:95)** (red curve).

**Figure S27.** DSC thermograms (5 °K/min) of the second heating scan of **PLU-b-PCL (15:85)** (blue curve), **PLU-b-PCL (10:90)** (green curve), and **PLU-b-PCL (5:95)** (orange curve).

**4. REFERENCES**

(1) Huang, Z.-Y.; Jiao, M.-R.; Gu, X.; Zhai, Z.-R.; Li, J.-Q.; Zhang, Q.-W. Asymmetric Synthesis of 1,2-Limonene Epoxides by Jacobsen Epoxidation. *Pharm. Front.* **2021**, *03* (03), e113–e118. https://doi.org/10.1055/s-0041-1740241.

(2) Delides, C.; Pethrick, R. A.; Cunliffe, A. V.; Klein, P. G. Characterization of Polyurethane Elastomers by 13C n.m.r. Spectroscopy. *Polymer (Guildf).* **1981**, *22* (9), 1205–1210. https://doi.org/10.1016/0032-3861(81)90133-6.
